# Building novel LLM-enabled explainable ensemble transformer models combining endoscopic and CT images for discriminating the different grades of gastrointestinal cancers

**DOI:** 10.3389/fmed.2026.1789769

**Published:** 2026-04-15

**Authors:** Ali Algarni, Ahood A. Al-Eidan, Surbhi B. Khan, Marwah A. Halwani, Ahlam Almusharraf, Yongwon Cho, Yunyoung Nam

**Affiliations:** 1 Department of Informatics and Computer Systems, College of Computer Science, King Khalid University, Abha, Asir, Saudi Arabia; 2 Center for Artificial Intelligence, King Khalid University, Abha, Asir, Saudi Arabia; 3 Department of Biology, College of Science, Imam Abdulrahman Bin Faisal University, Dammam, Saudi Arabia; 4 School of Science, Engineering and Environment, University of Salford, Manchester, United Kingdom; 5 Centre for Research Impact & Outcome, Chitkara University, Rajpura, Punjab, India; 6 Department of Management Information Systems, College of Business, King Abdulaziz University, Rabigh, Saudi Arabia; 7 Department of Management, College of Business Administration, Princess Nourah Bint Abdulrahman University, Riyadh, Saudi Arabia; 8 Department of Computer Science and Engineering, Soonchunhyang University, Asan, Republic of Korea

**Keywords:** cancers, ensemble transformer architectures, large language models, machine learning, user interaction

## Abstract

Gastrointestinal cancer (GC) is particularly malignant as they tend to progress slowly before advanced stages due to the forecast of early-specific symptoms. The heterogeneous properties of GCs require extremely precise and sensitive diagnostic techniques that can integrate in-depth structural information and surface-level data to distinguish cancer severity grades, thereby significantly lowering mortality rates. Deep learning (DL) algorithms are crucial in the classification of these various GC grades. However, the algorithms cannot detect interpretability and are characterized by a high probability of false alarm rates in detecting the underlying acute relationship between the medical images. Additionally, existing systems lack language-level transparency, preventing them from generating user-based narrative diagnostic explanations consistent with medical standards. To address this aforementioned challenge, this research study introduces a novel explainable LLM (X-LLM) based DL framework, which overcomes the drawbacks of existing DL algorithms. The suggested framework uses the ensemble transformer architectures that combine the clinical features by integrating the endoscopy and computer tomography (CT) scan images for enhancing the performance in detecting the different severity grades of GCs. The proposed system uses several components: (1) heterogeneous image collection; (2) image pre-processing; (3) ensemble networks; (4) interpretability analysis; and (5) user-interaction module. The extensive experiments are conducted utilizing two different datasets, such as Kvasir and TCIA CT (TCGA-STAD) scan images. The severity annotation of both datasets was carried out by experienced medical doctors, including endoscopists. Several evaluation metrics, including accuracy, precision, and recall, are measured and benchmarked against other learning networks. The experimental findings demonstrate the enhanced performance of the proposed framework over the existing models by achieving the accuracy, precision, recall, and F1-score values of 0.99, 0.997, 0.99, and 0.99, respectively. Furthermore, different LLM models such as GPT4.0, GPT3.5, LLAMA, and GEMINI are integrated, and their mode of interaction is also analyzed with SHAP measurements. The suggested framework demonstrates its strong potential by enhancing diagnostic performance, achieving high performance with user-interacted clinical treatment outcomes.

## 1 Introduction

Gastrointestinal cancers (GC) are mainly located in the gastrointestinal tracts and can be sub-divided into gastric cancers, esophageal cancers (EC), pancreatic cancer (PC), colon cancer (cc), and rectal cancer (RC) based on the cancer's location in the tracts (1–3). Nearly 35.4% accounted for increased mortality rates due to the GCs, followed by 26% increase due to the other gastro-related diseases. In 2022, more than 4,00,000 new cases and 3,00,000 deaths from the 5 spectrum of GC were recorded globally, which represents the importance of early prediction (4, 5). Furthermore, the mortality rate is expected to increase by 72%, resulting in more than 8 million new cases (6). The stages of GCs are categorized into very-low, low, moderate, and high-risk groups based on the references for prognosis defined by the 2008 National Institutes of Health (NIH) criteria (7). The statistics highlight the growing demand for the design of intelligent frameworks to assist healthcare centers in acquiring an accurate prediction of different stages of GC diagnosis. Addressing these challenges through the cognitive frameworks and endeavors becomes significant to effectively combat the rising burdens of GCs.

Recent innovations in artificial intelligence (AI) are implemented in designing the intelligent systems with reduced diagnostic errors related to traditional screening methods, thus improving overall detection accuracy (8–10). In this study, the Kvasir endoscopic dataset and the TCGA-STAD CT dataset, which originate from independent patient cohorts, were integrated at the feature level. Deep features extracted from both modalities were concatenated to enhance representation learning and improve the robustness of the proposed classification framework. In this context, deep learning algorithms and hybrid learning models are applied to accomplish the best clinical treatment process. However, AI-supported systems suffer from a significant challenge, which is the lack of clinical explainability, and are often regarded as non-transparent black-box algorithms. This limited visibility into how decisions are derived has created substantial barriers for healthcare facilities, particularly when utilizing the advanced and highly accurate clinical assessments, regardless of their performance (11, 12). Recent studies explored the implementation of explainable AI (XAI) techniques, such as SHAP (Shapley Additive Explanations), gradient-based attributions, and attention-based mechanisms, to enhance interpretability. However, existing XAI system integrates large language models (LLMs) to translate visual attention patterns, multi-modal embeddings, and cross-layer features into narrative clinical explanations consistent with radiology and endoscopy reporting standards (13–15). Without such language-level interpretability, the diagnostic process remains a “black box,” limiting transparency, accountability, and real-world adoption. Hence, the current AI system needs to be integrated with meaningful user-level interactions and high-level clinical trusts, making radiologists and gastroenterologists reluctant to rely on such models for high-stakes decision-making. In comparison to the existing methods of classification for gastric cancer, which are based on standalone CNNs or single-modal transformer models, the suggested methodology is an advancement in the SOTA as it combines ensemble transformer-based multi-scale feature extraction with structured temporal modeling and cross-modal attention fusion. In contrast to the existing methods that are only concerned with the accuracy of predictions, the proposed method combines LLM-driven interpretability to offer meaningful explanations along with the classification of severity. Additionally, the optimization provided by the QMPA-based method improves the stability of the model.

### 1.1 Motivation and contribution

To identify the previously noted factors shaping the decision-making process, this research outlines the novel and unique approaches in categorizing the severity grades of gastrointestinal cancers. The suggested framework incorporates the hybrid fusion of heterogeneous images, which can extract more clinical features to classify the severity grades in gastric cancers. The suggested model consists of the following components:

1) Heterogeneous data collection process: this component consists of different categories of images, such as Endoscopy and Computer tomography (CT) scan images, for extracting the high anatomical features revealing early-stage abnormalities that aid in the better classification of severity grades of GCs.
2) Ensemble transformer architecture module: the research introduces the novel ensemble transformer framework, which consists of dual sparse gated Swin transformer networks (DS-GST) for an effective feature extraction from different severity grades in the GCs.
3) Multi-feature fusion module: the novel multi-headed modal cross-attention layers are introduced to fuse the features from the different images for aiding better classification performances.
4) Quantum marine evoked extreme neural networks: the study introduces the QMENN for the better classification of endoscopic images with the severity grades.
5) LLM-based explainable module: finally, the study integrates an LLM-based interpretability mechanism to enhance clinical decision support. The LLM analyzes the model predictions along with Grad-CAM visualizations to provide meaningful textual explanations for the detected gastric cancer severity grades. This improves transparency in the decision-making process and assists clinicians in better understanding the rationale behind the classification outcomes.
6) Comprehensive experimentation: the comprehensive experiments have been conducted utilizing endoscopic images, and the evaluation metrics are analyzed.

### 1.2 Paper organization

The organization of the document is as follows: Section 2 summarizes the relevant literature contributed by various researchers. The suggested data collection, processing, segmentation framework, and optimized learning frameworks are detailed in Section 3. Section 4 provides the experimental outcomes, comparative analysis, and result discussion. Finally, Section 5 wraps up the research and provides the future improvement possibilities.

## 2 Related works

Ma et al. (16) proposed a deep learning fusion framework that incorporates the VGG16, ResNet-50, and MobileNetV2 architectures to detect gastric cancer (GC) using the GasHisSDB dataset. Their approach used dependable feature extraction and extensive contextual understanding from histopathological images, enhanced with the LIME technique for transparency. The recommended approach attained an accuracy of 97.8%, which was 7% better than standalone models, and the LIME visualizations showed important parts of the image that help in decision making on cancer detection. Although the study utilizes a single dataset (GasHisSDB), this can limit the applicability in various clinical environments and scan quality.

Huang et al. (17) developed a radiomics-driven machine learning (ML) approach utilizing CT images to identify major pathological response (MPR) in locally advanced GC patients receiving neoadjuvant immunotherapy. They conducted a multicenter study that involved 268 patients in development (*n* = 86), internal validation (*n* = 59), and external validation (*n* = 52) cohorts, using nine Machine Learning algorithms, with Bayesian-LightGBM chosen as the best. The framework attained AUC values of 0.828, 0.777, and 0.714 in the three cohorts and a general accuracy of 0.791, with SHAP analysis showing the key radiomic features in making the prediction. Despite the high generalizability of the model, its moderate accuracy can limit its use in clinical settings, particularly in centers with diverse imaging practices or equipment variations.

Yao et al. (18) evaluated three locally deployed LLMs (INF-72B, Qwen2.5-72B, and LLaMA3.1-70B) to automatically identify EC stages using 1,134 Chinese free-text radiology records and compared their efficiency with that of medical practitioners. The study presented an interpretable reasoning (IR) prompting strategy, and INF-72B+IR reached 61.5% overall staging accuracy and 0.60 F1-score, which is significantly higher than 39.5% and 0.39 F1-score of clinicians. Qwen2.5-72B+IR also performed better (46% accuracy), whereas LLaMA3.1-70B showed no significant difference compared to medical experts. The relatively low absolute accuracy rates (maximum of 61.5%), however, indicate that the models still need to be refined before they can be used on a clinical basis.

Wan et al. (19) proposed DeepGut, a multimodal collaborative LLM model with a four-tier structure for digestive disease diagnosis. The system extracts multimodal information (Tier 1), constructs a logical diagnostic chain (Tier 2), offers diagnostic and treatment suggestions (Tier 3), and analyzes risks (Tier 4), combining patient history, laboratory findings, and images. DeepGut framework showed superior performance with 97.8% diagnostic accuracy, 93.9% diagnostic completeness, 95.2% treatment plan accuracy, and 98.0% treatment plan completeness, but showed reduced practical clinical performance because of greater input/output token counts.

Khayatian et al. (20) designed a hybrid ML model involving both the EfficientNetV2B0 model and CatBoost classifier for the categorization of gastric histopathology images, which relied on the GasHisSDB dataset. Their algorithm delivered accuracies of 89.7, 93.1, and 93.9% with 80, 120, and 160 px image cropping sizes, respectively. Grad-CAM visualization confirmed that the model focused on histology-specific regions, and t-SNE visualization revealed clear separations between normal and diseased samples after extracting features. Although well-performing, the analysis relied on one dataset, which might limit its application to different clinical settings.

Zubair et al. (21) created an interpretable model based on multi-channel attention and transfer learning for gastric cancer classification. They used three parallel attention channel mechanisms alongside CNN to extract features in multiple channels. The research achieved high recognition rates of 99.07 and 98.48% on the validation and test partitions of the gastric histopathology sub-size image collection, and 99.84%, 99.65% accuracies when tested on the HCRF dataset. Grad-CAM-based heatmaps provided a good interpretability capability, recognizing the most important parts of the image that guided the model prediction process. Nonetheless, the study did not cover any computational analysis, and it is unclear how effective and practical such a model would be when used in a real clinical application.

Zhang et al. (22) presented GutGPT, a dedicated LLM that relied on Baichuan-13B-Chat and optimized with the help of Low-Rank Adaptation (LoRA) and shared-parameter self-attention modules to provide state-of-the-art applications to gastrointestinal healthcare. The system was trained with a curated QA corpus with 191,611 items, including clinical dialogues, medical knowledge networks, medical practice guidelines, and materials on professional licensing examinations. GutGPT improved the performance of diagnostic results by 9.59% relative to benchmark systems grounded on specialist assessment and attained an average accuracy of 22.47% with the CMB and CMExam public dataset, thus showing good accuracy and strong empathy. However, the study lacked an external clinical validation in actual hospital settings, and its applicability and reliability in the real world remain unclear.

Qin et al. (23) designed a hybrid data-driven architecture that incorporates LLM-based chatbots with endoscopic image data and patient-level clinical information to enhance the accuracy of gastrointestinal diagnostics. The system uses hierarchical feature integration, attention-enhanced decision making, and a cross-domain learning mechanism in order to guarantee high diagnostic effectiveness. The algorithm was tested on Kvasir-SEG, GastroVision, MIMIC-IV, and CMU-MOSEI datasets and attained 88.2, 83.7, 92.1, and 90.3% accuracies, respectively. The system enhanced lesion identification, minimized the analysis variability, and improved the integration between clinicians and AI. Although these findings, the LLM-based chatbot subsystem was not experimentally tested but was left as an idea, which restricted the potential evidence regarding its practical clinical utility.

Binzagr (24) designed an XAI-based GC classification model based on a hybrid ensemble of three CNNs (InceptionV3, InceptionResNetV2, and VGG16) with SHAP methods to interpret the results. The combined model, trained on the KvasirV2 dataset of pathological cancer symptoms, achieved 93.17% accuracy and 97% F1-score, respectively, and SHAP analysis offers visual explanations of prediction choices. Nonetheless, the assessment of the framework was based on one dataset (KvasirV2), which does not reflect the whole range of gastrointestinal pathologies.

Mudavadkar et al. (25) suggested an ensemble learning approach for the detection of GC on digital pathology utilizing the GasHisSDB dataset. Their stacking architecture used ResNet34 and VGGNet16 as baseline models, which attained more than 99% average accuracy on different image resolutions, and ResNet50, VGGNet, and ResNet34 models also outperformed EfficientNet or VitNet. The proposed method was effective in identifying key characteristics of smaller image patches, which also indicates the possibility of early diagnosis. Nevertheless, the reported accuracy of the recommended model (>99) on single datasets is extremely high, which might show a possibility of overfitting or inadequate testing on difficult edge cases.

Table 1 summarizes the distinct algorithms adopted in related research works, highlighting their key findings and limitations.

**Table 1:** Related work's summary.

S. No	Author	Algorithm	Findings obtained	Advantage	Limitation
1	Ma et al. (16)	DL Fusion framework (VGG16 + ResNet-50 + MobileNetV2) with LIME	Accuracy: 97.8% (7% improvement over standalone models)	Robust feature extraction with explainability through LIME visualizations	Reliance on a single dataset (GasHisSDB) limits generalizability across diverse clinical settings
2	Huang et al. (17)	Bayesian-LightGBM Model	Overall accuracy: 0.791	Strong generalizability across multicenter cohorts with SHAP analysis	Moderate accuracy may constrain clinical adoption in heterogeneous imaging protocols
3	Yao et al. (18)	LLMs (INF-72B, Qwen2.5-72B, LLaMA3.1-70B) with interpretable reasoning prompting	INF-72B+IR model achieved a high accuracy-61.5% among the other two models	Automated esophageal cancer staging from free-text reports with interpretable reasoning	Modest absolute accuracy (61.5% maximum) requires further refinement for clinical deployment
4	Wan et al. (19)	DeepGut Approach- Multimodal LLM with four-tiered architecture	Achieved 97.8% diagnostic accuracy	Comprehensive collaborative framework integrating multimodal data for diagnosis and treatment	Substantially increased computational costs and extended diagnostic times limit real-time clinical utility
5	Khayatian et al. (20)	Hybrid Model- EfficientNetV2B0 for Feature Extraction, CatBoost for Classification	Accuracy: 89.7, 93.1, 93.9% on 80, 120, 160 px images respectively	Grad-CAM visualization for focused learning; t-SNE demonstrated clear clustering	Single dataset reliance (GasHisSDB) limits generalizability to diverse clinical settings
6	Zubair et al. (21)	Multi-channel attention mechanisms with transfer learning	Accuracy: validation: 99.07%, Testing: 98.48% on GasHisSDB; validation: 99.84%, Testing: 99.65% (HCRF dataset)	Interpretable framework with Grad-CAM visualizations highlighting decision-making regions	No computational analysis provided
7	Zhang et al. (22)	Baichuan-13B-Chat LLM fine-tuned with LoRA.	9.59% improvement over baseline models	High accuracy and strong empathy in gastrointestinal disease management	Lacks external clinical validation from real hospital settings
8	Qin et al. (23)	Multimodal learning framework with LLM chatbots	Accuracy: 88.2, 83.7, 92.1, 90.3% on Kvasir-SEG, GastroVision, MIMIC-IV, CMU-MOSEI datasets, respectively	Multi-scale feature fusion with attention-guided diagnosis; enhanced physician-AI collaboration	LLM-based chatbot component remained conceptual without experimental validation
9	Binzagr (24)	Hybrid ensemble CNNs (InceptionV3 + InceptionResNetV2 + VGG16) with SHAP	Accuracy: 93.17%, F1-score: 97%	Explainable AI with SHAP, providing visual explanations of prediction decisions	Evaluation limited to a single dataset (KvasirV2) may not represent the full spectrum of GI pathologies
10	Mudavadkar et al. (25)	Stacking ensemble approach (ResNet34 + VGGNet16)	Average accuracy: >99% across various image resolutions	Successful feature extraction from smaller image patches for early diagnosis	Extremely high accuracy on a single dataset may indicate potential overfitting or insufficient edge case evaluation

## 3 Proposed methodology

**Figure 1:**
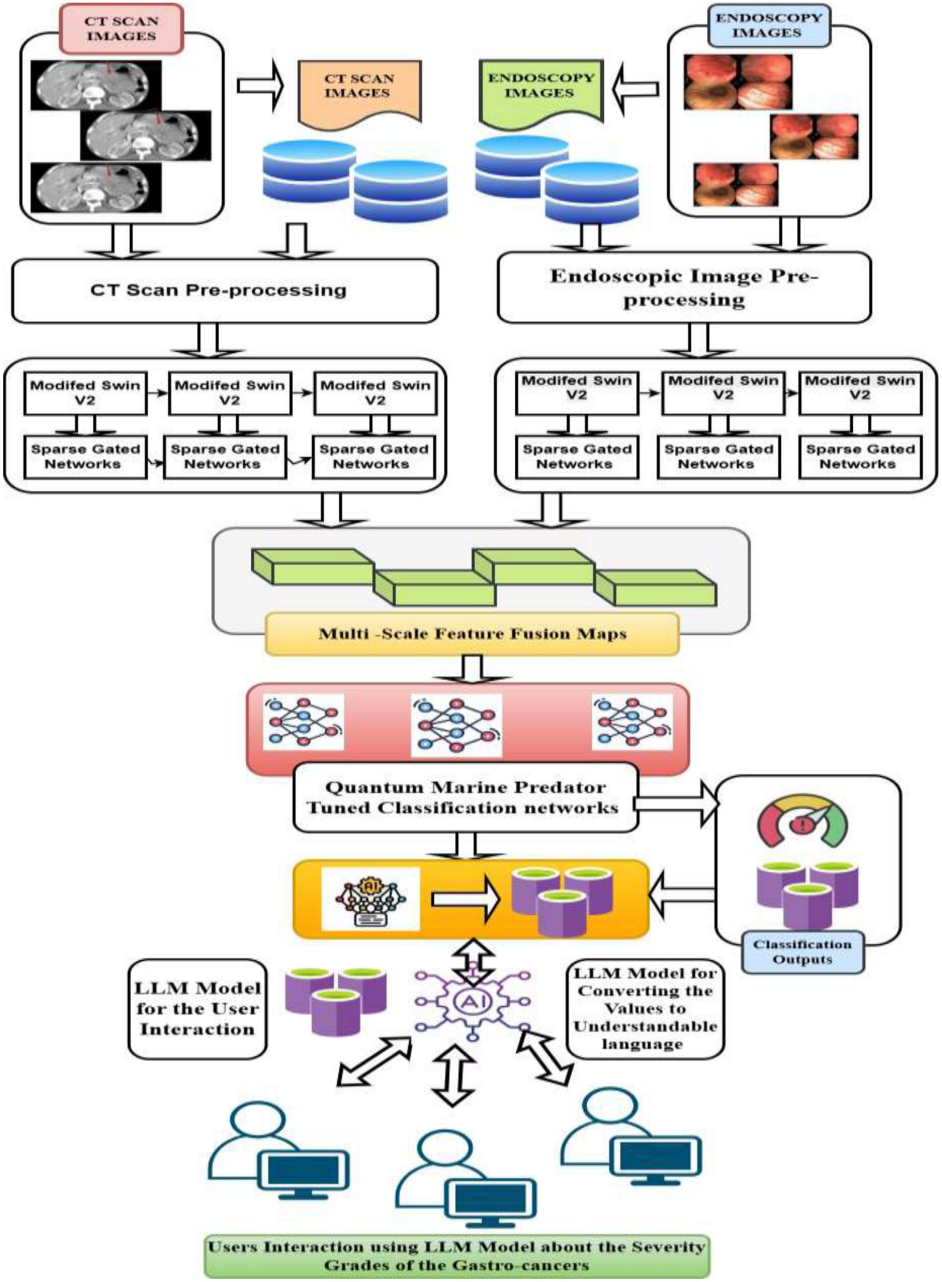
Overall systematic architecture for the proposed system.

The suggested schemes deliberately present an LLM-based XAI-driven framework for gastrointestinal cancer detection. Figure 1 demonstrates the suggested framework of X-LLM driven intelligent systems for the classification of severity grades in GCs. The model was trained and assessed utilizing the different datasets to uncover the most important anatomical features that have the potential to enhance the performance of detection. Additionally, the LLM-enabled XAI framework was employed to identify the key contributors linked to each category.

### 3.1 Dataset description

**Figure 2:**
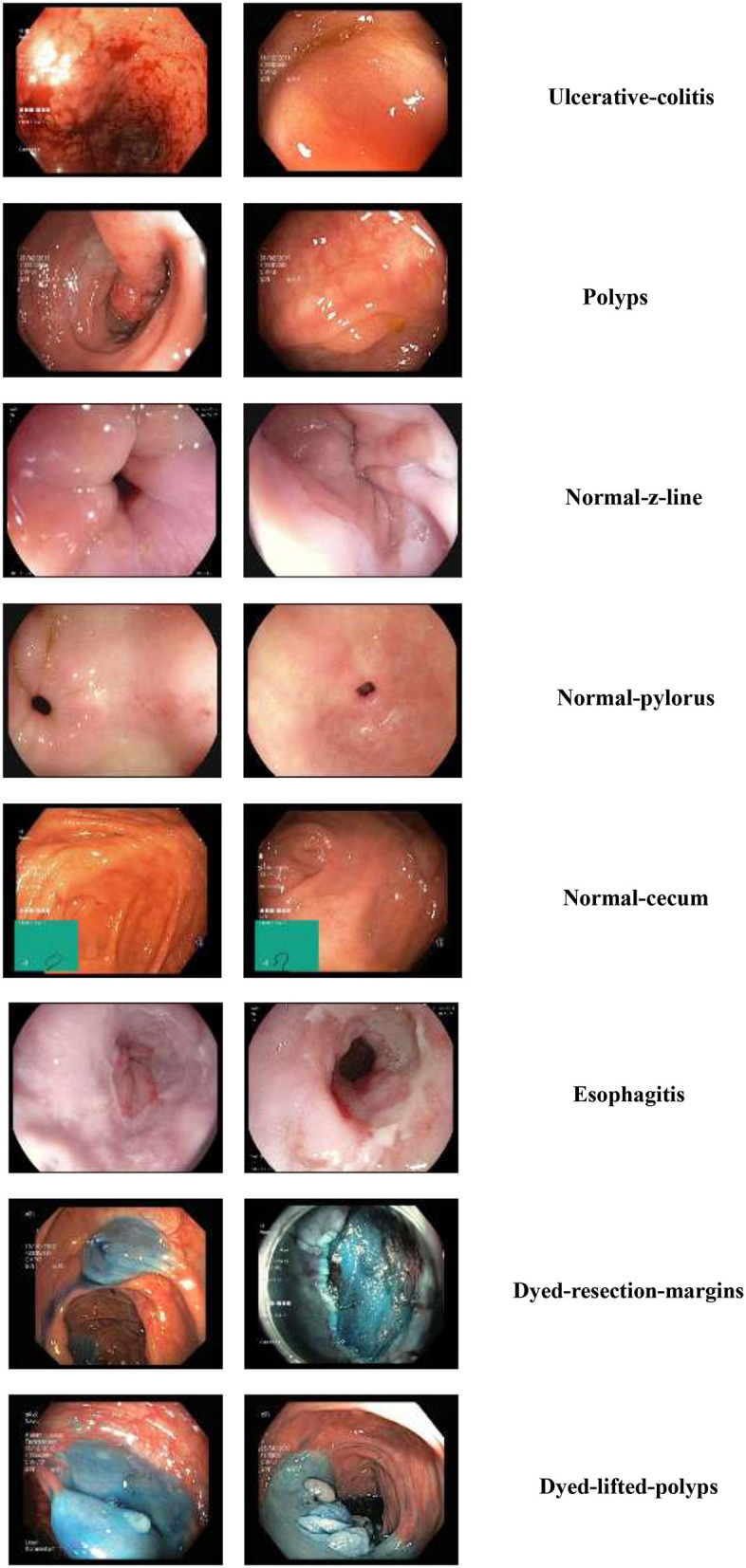
Sample endoscopic images with the variable labels.

As shown in Figure 2, the Kvasir dataset (26) is one of the most widely used GC endoscopy image collections designed for early detection of GC disorders and is utilized for the research. The datasets contain more than 8,000 annotated endoscopic images distributed across multiple clinically relevant classes, including polyps, esophagitis, ulcerative lesions, esophagus, normal mucosa, dyed-lifted polyps, and anatomical landmarks. Each image is captured using high-resolution cameras suitable for the real-time clinical environment. Figure 2 shows the sample images used for the training and evaluation process. These characteristics make Kvasir an ideal benchmark for training and evaluating models that require fine-grained feature extraction, especially those targeting early-stage lesions or subtle mucosal changes indicative of GI cancers.

**Figure 3:**
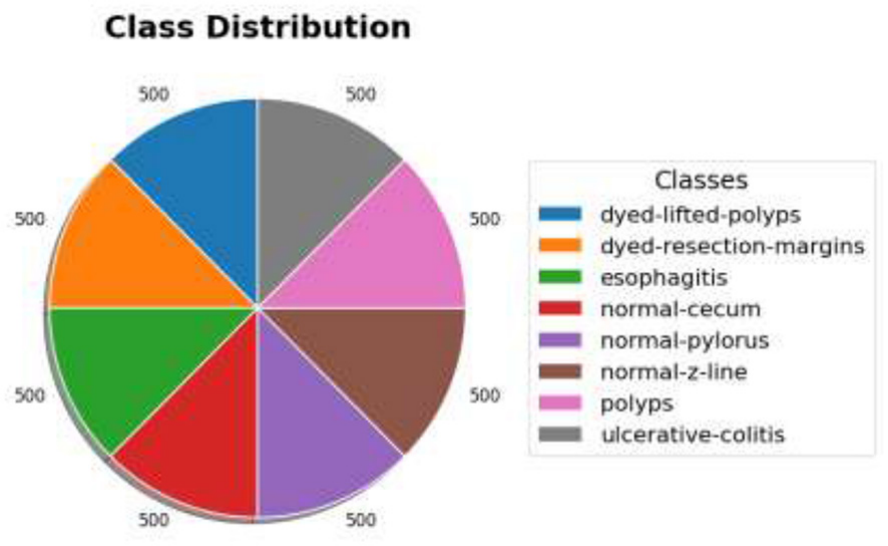
Class distribution process for the Kvasir datasets utilized for training and evaluating the model.

In the second stage of the dataset, CT-based gastro images obtained from the TCGA-STAD were utilized to fuse with the endoscopic images to increase the diversity of feature representations. The CT data set has 214 patients with contrast-enhanced abdominal CT scans available. The volumetric scans were transformed into two-dimensional axial slices, and approximately 32,000 CT images were used for training and validation. The severity labeling of both data sets was done and validated by medical professionals, including experienced endoscopists, to ensure that the labeling is reliable for diagnosis. Figure 3 presents the class distribution process in both datasets. From Figure 3, it is evident that there is no significant class imbalance problem, thereby reducing the risk of overfitting and improving classification performance.

### 3.2 Data pre-processing

For an efficient pre-processing strategy, CLAHE has been employed to elevate the contrast variations within the endoscopic and CT scan images. After increasing the contrast levels, all input images are scaled to 244 × 244 to facilitate efficient feature extraction and classification. Prior to any augmentation or pre-processing operations, the complete dataset consisting of approximately 40,000 images (8,000 endoscopic and 32,000 CT images) was split into three mutually exclusive subsets: training (70%), validation (10%), and evaluation (20%). This resulted in 28,000 real images allocated for training, 4,000 images for validation, and 8,000 images for testing. This splitting was done before the training of the models to ensure that none of the images in the validation or test sets were used during the training process. Following contrast enhancement and resizing, image augmentation was applied exclusively to the training subset to address dataset imbalance and improve generalization. In this research, the StyleGAN2 model (27) was adopted to mitigate overfitting by generating closely related synthetic endoscopic images. In contrast to the conventional augmentation techniques like rotation, flip, and crop, which are only applied to existing images, StyleGAN2 is capable of producing new and realistic samples of endoscopic images by learning the underlying data distribution. This increases the diversity of pathological samples and prevents overfitting, as evident from the excellent FID, PSNR, and SSIM scores that show high similarity between real and generated images. A total of 5,600 synthetic samples were generated and added only to the training subset, increasing the final training dataset size to 33,600 images. The quality of the generated synthetic endoscopic samples was quantitatively evaluated by comparing real and synthetic images using FID, PSNR, and SSIM. The obtained results (FID = 23.8, PSNR = 34.7 dB ± 1.2 dB, SSIM = 0.89 ± 0.03) indicate high perceptual similarity, strong structural consistency, and low distributional divergence between the generated and real endoscopic images. The validation and testing sets consist entirely of real, unseen images and were not exposed to synthetic samples. Algorithm 1 presents the complete augmentation process using the StyleGAN2 model.

Algorithm 1 Working mechanism of the Suggested StyleGAN2 Model.

**Step**
1 **Input :** Input Images I(w) with dimension =
  224 × 224
2   Image Resolution I(R) with 512
3 **Output :** Endoscopic Image Generations S(w)
4 Initialize the Suggested Networks with
  Generator (G), Discriminator (D) and
  Mapping (M) with I(w) and I(R)
5     Optimize the Networks using ADAMw
6 **For n** **=** **1: max_training_loop**
7     Real_Images = datasets_Images
8     Generated image S(w) = D(M(I))
9     Save (Generated Images)
10    Calculate the Discriminator Loss D(L)
      = loss(S(w))
11    Back propagate and optimize the
      networks with D(L)
12     Apply Regularization on the S(w)
13     Add the S(W) in the I(w)
14 **End**

### 3.3 Model design

The following section details the proposed Swin Transformer Framework, Sparse Gated Recurrent Units, and the suggested model.

#### 3.3.1 Swin V2 model design

**Figure 4:**
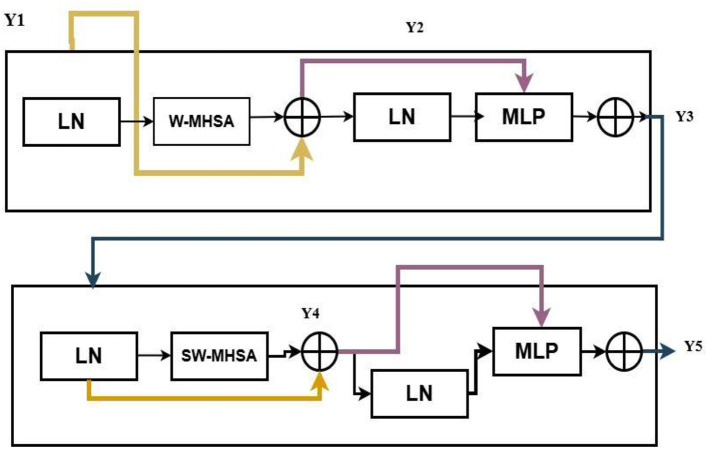
Swin V2 model—its model architecture.

SwinV2 Model is a modified version of the hierarchical transformer architecture, which is used to overcome the limitations of traditional Swin models for handling high-resolution medical imaging tasks. The suggested SwinV2 model contains several architectural refinements in the traditional Swin transformers, which include the post-normalization and logit-scale attention, within local non-overlapping windows. By evaluating self- attention within local non-overlapping windows and periodically shifting windows across layers, Swin V2 accomplishes a robust trade-off between comprehensive contextual comprehension and computational performance. This hierarchical windowing approach allows the algorithm to derive multi-scale representations, and it is especially effective in capturing fine-scale mucosal textures as well as large-scale structural patterns that are often present in gastrointestinal images. Moreover, SwinV2 utilizes Log-spaced continuous position Bias (Log-CPB) to provide an efficient mechanism to transfer models trained on lower-resolution inputs to higher-resolution downstream applications. It also uses the self-supervised pre-training SimMIM strategy, which does not rely on large annotated datasets. Figure 4 gives the architecture of Swin Transformer V2 (STV2). This module comprises two major units: The Window-based Multi-Head Self-Attention (W-MSA) mechanism and the Shifted Window Multi-Head Self-Attention (SW-MSA) mechanism.

The ST V2 primarily operates through its window-based self-attention mechanism. The pixel regions inside each window are reshaped by compressing their spatial dimensions to create a two-dimensional feature matrix. This representation is then used to derive the query (Q), key (K), and value (V) tensors required for attention processing. These Q, K, and V tensors are evenly split according to the number of attention heads h. Each portion is linearly mapped through h independent projection layers, enabling parallel multi-head attention computation. The outputs generated by all heads are combined through concatenation and passed through a final linear projection to produce the refined output features shown in Equations 1–4.


$$\begin{array}{l} Y1=W-MHSA\left(LN\left(y1\right)\right)+\left(Y1-Y2\right) \end{array}$$ (1)

$$\begin{array}{l} Y3=MLP\left(y1\right)+\left(y1\right) \end{array}$$ (2)

$$\begin{array}{l} Y4=SW-MHSA\left(LN\left(Y3\right)\right)+\left(Y3+Y4\right) \end{array}$$ (3)

$$\begin{array}{l} Y5=MLP\left(Y4\right)+\left(Y4\right) \end{array}$$ (4)

#### 3.3.2 Modified dilated residual Swin V2 model

Despite the advantages of the SwinV2 model, it needs several updates in handling the endoscopic images in terms of varying the local-window attention maps to handle the fully captured long-range dependencies across highly irregular endoscopic anatomical structures, which often contain elongated folds and curved mucosal surfaces. Additionally, attention layers may overfit to low-level features when exposed to the heterogeneous color profiles of images. To overcome this challenge, this research introduces the Multi-Headed Dilated Residual Attention maps in the Swin V2 Model to facilitate the multi-scale feature extraction process in accordance with the endoscopic images.

##### 3.3.2.1 Multi-headed dilated residual attention maps

**Figure 5:**
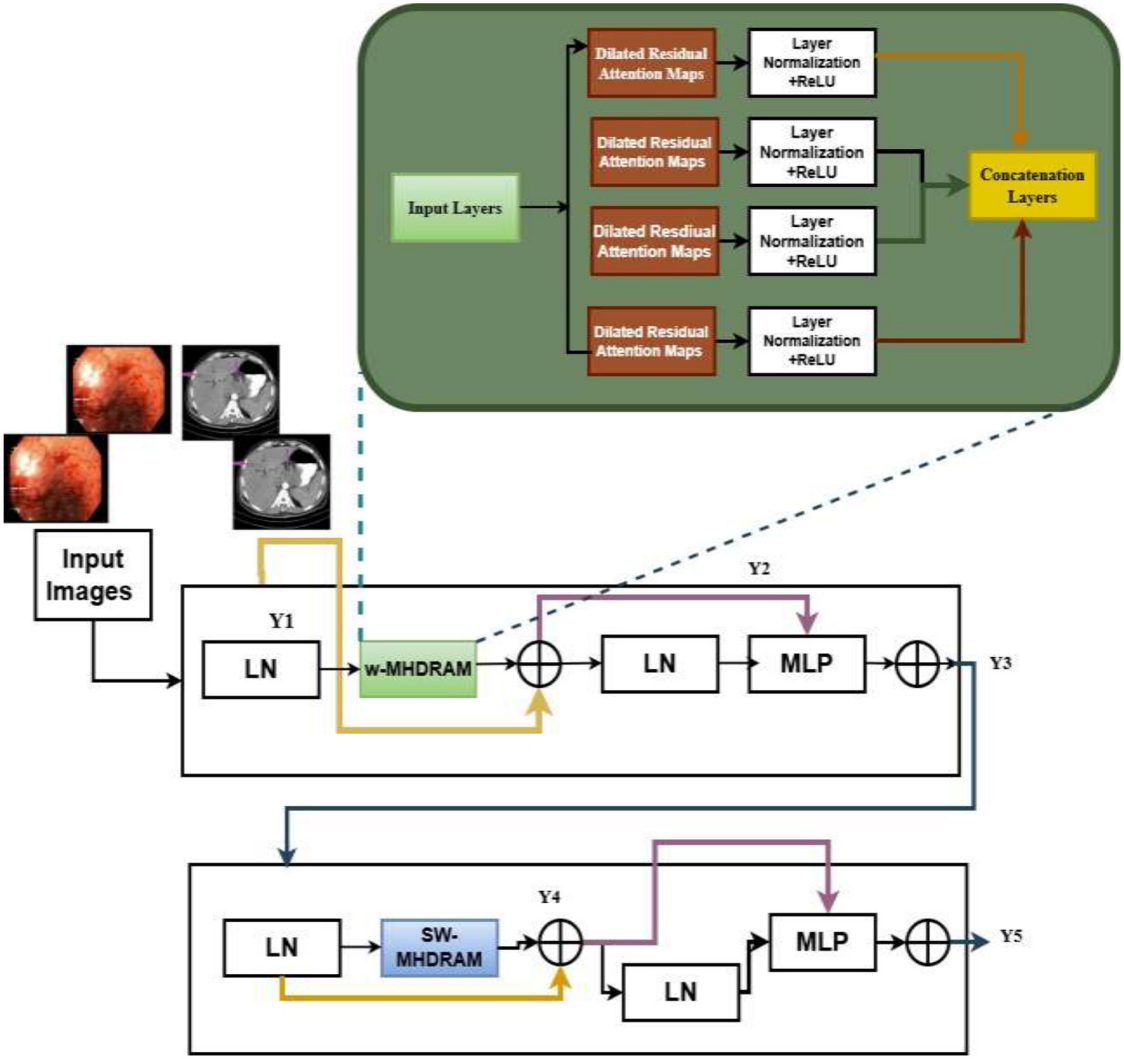
Overall architecture for the modified Swin V2 model with the MHDRAM layers.

To enhance the model's stability, this research introduces the Multi-Headed Dilated residual attention maps (MHDRAM) as an advanced attention mechanism integrated into the SwinV2 structure. The suggested MHDRAM introduces the dilated residual convolutional networks with multiple dilation rates into each attention layer, enabling the networks to acquire both fine-grained mucosal textures and large anatomical variations simultaneously. The suggested architecture consists of four layers of MHDRAM, in which each attention head processes at different dilation scales, enabling effective capturing of irregular and complex GC surface patterns that vary across the endoscopic images. Each layer is preceded by batch normalization and an activation (ReLU) layer to overcome the overfitting problems. Finally, the output of these dilated attention heads is concatenated to form the adaptive feature maps, ensuring stable gradient propagation and preserving salient low-level features while preserving high-level contextual patterns. Figure 5 depicts the complete structure of the Modified Swin V2 framework integrated with MHDRAM layers to enhance feature extraction and classification performance.

#### 3.3.3 Sparse gated networks

Sparse GRU is regarded as one of the notable types of Gated Recurrent Units (GRU). This concept was designed by Chung et al. (28) and is intended to combine the forget gate and input vector within a single homogeneous vector. This model can conveniently deal with long-term sequences and persistent memory and is much simpler to compute than long short-term memory (LSTM) networks.

The equations formulated by Chung represent the core operations of the GRU, which are as follows in the Equations 5–8:


$$\begin{array}{l} h_{t}=\left(1-x_{t}\right)\;⊙\;h_{t-1}+x_{t}\;⊙{\;h}_{t} \end{array}$$ (5)

$$\begin{array}{l} \tilde{h_{t}}=g\left(W_{h}x_{t}+\;U_{h}\left(r_{t}\;⊙\;h_{t-1}\right)+\;b_{h}\right) \end{array}$$ (6)

$$\begin{array}{l} z_{t}=\sigma \left(W_{h}x_{t}+U_{z}h_{t-1}+\;b_{z}\right) \end{array}$$ (7)

$$\begin{array}{l} r_{t}=\sigma \left(W_{h}x_{t}+U_{r}h_{t-1}+\;b_{r}\right) \end{array}$$ (8)

Here *x*_*t*_ signifies the input feature at the current time step, *y*_*t*_ indicates the output state, *h*_*t*_ represents the hidden state of the module at the current time step, *Z*_*t*_ and *r*_*t*_ correspond to the update and reset gates, *W*(*t*) signifies the weight parameters, and *b*(*t*) signifies the bias at the current time step.

#### 3.3.4 Suggested sparse GRU network

In the proposed research, sparse matrices are introduced in the GRU networks to reduce the computation of the networks, which can be suitable for a latency-free learning process. The suggested sparsity concept refers to zeroing entire blocks of the weight matrices rather than individual weights. This approach allows for better optimization of weights suitable for extracting the temporal features from the gastro images. The sparse weights are given in Equation 9:


$$\begin{array}{l} Sparse\;Weights\;;\;W^{\prime \prime }=\;W\;⊙S \end{array}$$ (9)

where *M* is a binary mask matrix of the same shape as *W*. Hence, Equations 5–8 are replaced with the above equation, and the sparse GRU networks' characteristics are formulated as follows in Equations 10–13:


$$\begin{array}{l} h_{t}=\left(1-x_{t}\right)\;⊙\;h_{t-1}+x_{t}\;⊙{\;h}_{t} \end{array}$$ (10)

$$\tilde{h_{t}}=g\left({W^{\prime \prime }}_{h}x_{t}+\;U_{h}(r_{t}\;⊙\;h_{t-1})+\;b_{h}\right)$$ (11)

$$\begin{array}{l} z_{t}=\sigma \left({W^{\prime \prime }}_{h}x_{t}+U_{z}h_{t-1}+\;b_{z}\right) \end{array}$$ (12)

$$\begin{array}{l} r_{t}=\sigma \left({W^{\prime \prime }}_{h}x_{t}+U_{r}h_{t-1}+\;b_{r}\right) \end{array}$$ (13)

The introduction of the Sparse matrix in GRU networks will consume less computation as compared with the other three variants discussed in (29). The suggested Sparse GRU facilitates adaptive cross-modal fusion when combined with endoscopic features, as the recurrent gates dynamically weigh adaptively in accordance with texture-rich endoscopy and structure-rich CT imaging.

### 3.4 Encoder–decoder architecture

**Figure 6:**
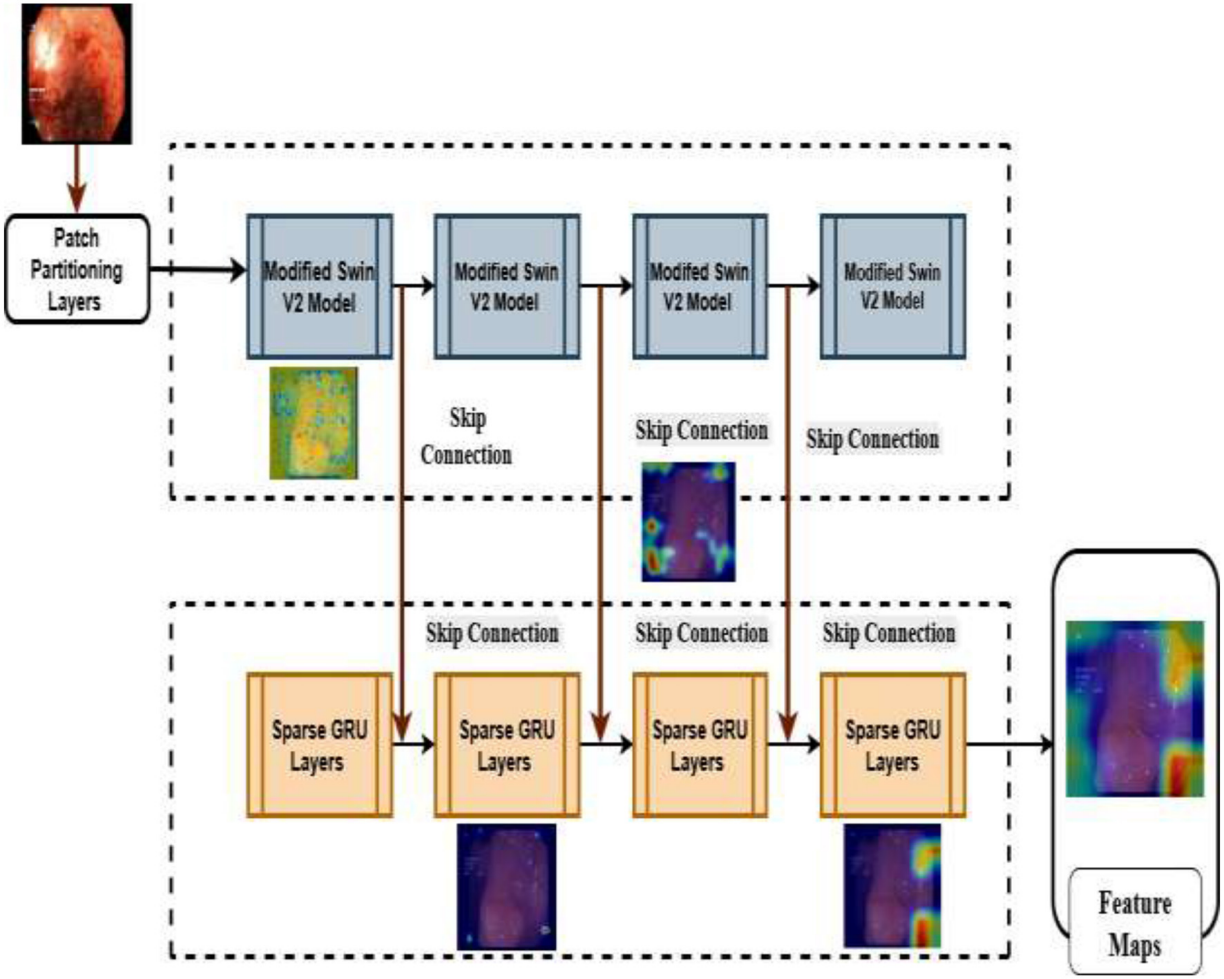
Encoder–decoder architecture for the suggested framework with the intermediate layered outputs.

As shown in Figure 6, the encoder structure of the proposed framework consists of four layers of the SwinV2 model with MHDRAM structures. Endoscopic visuals are initially introduced into the patch partition layers to create sequences, which are then encoded by linear embedding layers. After that, the projected image patches move through the four-layered SwinV2 variant blocks and patch fusion layers to construct layered feature maps.

As depicted in Figure 6, the decoder of the suggested framework comprises four hierarchical layers. Each layer incorporates a Sparse Gated unit with the encoder to extract spatio-temporal features. Moreover, patch expansion modules are employed to gradually enhance spatial resolution while simultaneously reducing channel depth for optimized upsampling. The decoder further processes the extracted features across subsequent layers (*i* = 3, 2, 1) by leveraging the Sparse GRU networks. Finally, the attention maps are used to concatenate the features from the encoder–decoder at the high-resolution process. Furthermore, patch expanding layers are added in the final layers to restore the feature map to the original image resolution to enable pixel-level prediction. The suggested framework combines the sparse GRU networks combined with the modified Swin transformer for extracting the multi-scale, multi-modal, and sequential features, enabling highly accurate and highly resilient performances in detecting the different grades of the gastro cancers.

### 3.5 Modality cross-attention fusion layers

The proposed research incorporates Multi-Scale Modality Cross-attention Maps (MSMCAM) to explicitly explores the complementary relationship between endoscopic and CT gastro images, enabling an effective relationship between the structural mucosal features and the anatomical mechanism. modality cross-attention computes the interaction between two heterogeneous modalities by using one modality's features as queries and the other modality's features as keys and values. This allows the network to identify which regions in endoscopic images correspond to structural abnormalities observed in CT slices, which helps in identifying the severity grades in each class of endoscopic images. Mathematically, the functionalities of feature extraction maps are expressed as follows in Equation 14, 15:


$$\begin{array}{l} F\left(Fusion\text{\_}Endoscopic\right)=\\ \;Softmax\left(\left(\left(K\right),Q^{T\left(endoscopic\right)}\right)\right)/{\left(V_{K}\right)}^{\wedge }0.5 \end{array}$$ (14)

$$\begin{array}{l} F\left(Fusion\text{\_}CT\right)=Softmax\left(\left(\left(K\right),Q^{T\left(CT\right)}\right)\right)/{\left(V_{K}\right)}^{\wedge }0.5 \end{array}$$ (15)

Finally, the final feature maps are obtained by concatenating both features using Equation 16:


$$\begin{array}{l} Total\;Features\;F\left(Fused\right)=\;Concat\;(F\left(Fusion_{Endoscopic}\right),\\ \;F\left(Fusion_{CT}\right)) \end{array}$$ (16)

### 3.6 Quantum marine evoked dense networks

This section details the deep feed-forward networks in which the hyper-parameters are fine-tuned by the quantum marine predator algorithm (QMPA). A comprehensive explanation of each module is provided below.

#### 3.6.1 Quantum marine predator algorithm

The marine predators algorithm (MPA), categorized as a population-based optimization method, shares similarities with several other metaheuristic techniques. A notable attribute of this algorithm is its periodic dispersion of the starting solution throughout the search domain, primarily for illustrative purposes, as articulated in Equation 17.


$$\begin{array}{l} S_{0}=S_{\min}+rand\left(S_{\max}-S_{\min}\right) \end{array}$$ (17)

Rand is an indicator of a random vector that has values between 0 and 1, where *S*_min_ and *S*_max_ denote as the minimum and maximum of the parameters being optimized. This preliminary propagation of solutions forms the base for the following stages of optimization. Within the framework of the Marine Predators algorithm, the most optimal solution found is called the dominant predator, which plays a key role in creating a matrix called Elite, as shown in Equation 18. These matrices play a crucial role in tracking and identification of prey, utilizing their position coordinates efficiently.


$$\begin{array}{l} Elite={\left[\begin{array}{c} S_{1,1}^{I},S_{1,2}^{I},...S_{1,d}^{I}\\ S_{2,1}^{I},S_{2,2}^{I},...S_{2,d}^{I}\\ .............\\ S_{n,1}^{I},S_{n,2}^{I},...S_{n,d}^{I} \end{array}\right]}_{n\times d} \end{array}$$ (18)

The Elite matrix, $S_{n,d}^{I}$ denotes the vector corresponding to the optimal predators, where *n* is the predator count, and d is the dimensionality. This matrix changes dynamically when a better agent beats the existing optimal predators. Similarly, the Prey matrix has the same dimensional structure as the Elite matrix. Prey-seeking agents change their positions with the data present in the Prey matrix. Subsequently, the initializing stage is significant in shaping the preliminary Prey matrix, since the Elite matrix is generated on the basis of the most successful predators. The Prey matrix is formulated in Equation 19.


$$\begin{array}{l} Prey={\left[\begin{array}{c} S_{1,1},S_{1,2},...S_{1,d}\\ S_{2,1},S_{2,2},...S_{2,d}\\ .............\\ S_{n,1},S_{n,2},...S_{n,d} \end{array}\right]}_{n\times d} \end{array}$$ (19)

The interaction and dynamic nature of the Prey and Elite matrices is the underlying force behind the optimization process of the MPA, which reflects its unique problem-solving approach. This procedure is organized into three different stages, which altogether constitute the MPA approach in seeking the best solutions.

##### 3.6.1.1 Stage 1—High-velocity ratio (HVR)

At this phase, the prey is slower than the predators, and exploration is critical in the initial optimization stages. The numerical representation of this step is given by:

*While Iteration* < *1/3* × *max (Iteration)*


$$\begin{array}{l} \begin{array}{c} stepsize_{k}=M_{B}⊗\left(Elite_{k}-M_{B}⊗Prey_{k}\right),k=1,\ldots ,n\\ \;Prey_{k}=Prey_{k}+P\cdot M⊗stepsize_{k} \end{array} \end{array}$$ (20)

The term *M*_*B*_ represents the Brownian motion that is modeled on a uniformly distributed random vector. Symbol ⊗ indicates element-wise multiplication, which emulates the prey motion by incorporating. *M*_*B*_ to the computation. There are two elements in this phase: a constant P and a vector M of random values that are evenly distributed between 0 and 1.

##### 3.6.1.2 Stage 2—Uniform velocity ratio (UVR)

In this stage, predators and prey move at equal velocities, and there must be a balance between the exploration and exploitation strategies. During this stage, half of the agents are allocated to exploratory tasks, and the other half should concentrate on exploitation. Preys and predators work together to complete these two roles. The mathematical description of this step is shown below in Equation 21.

*While 1/3* × max*(Iteration)* < *Iteration* < *2/3* × max*(Iteration)*


$$\begin{array}{l} stepsize_{k}=M_{L}⊗\left(Elite_{k}-M_{L}⊗Prey_{k}\right),k=1,\ldots ,n/2\\ \;Prey_{k}\;=Prey_{k}+P\cdot M⊗stepsize_{k} \end{array}$$ (21)

The movement of prey is modeled by generating the vector, *M*_*L*_ which uses the Levy Flight (LF) mechanism, incorporating the position of prey with an estimated step size. According to the MPA, the remaining 50% of the population adheres to the behavioral principles described in Equation 22.


$$\begin{array}{l} stepsize_{k}=M_{B}⊗\left(M_{B}⊗Elite_{k}-Prey_{k}\right),k=n/2,\ldots ,n\\ \;Prey_{k}=\;Elite_{k}+P\cdot CF⊗stepsize_{k}\\ \;CF\;={\left(1-\frac{Iteration}{Max\left(Iter\right)}\right)}^{2\times \frac{Iteration}{Max\left(Iter\right)}} \end{array}$$ (22)

##### 3.6.1.3 Stage 3—Low-velocity ratio (LVR)

This stage occurs when the prey possesses a lower velocity relative to the predators, which is explained in Equation 23:

*While Iteration* > *2/3* × *max (Iteration)*


$$\begin{array}{l} stepsize_{k}=M_{L}⊗\left(M_{L}⊗Elite_{k}-Prey_{k}\right),k=1,\ldots ,n\\ \;Prey_{k}=Elite_{k}+P\cdot CF⊗stepsize_{k}\\ \;CF={\left(1-\frac{Iteration}{Max\left(Iter\right)}\right)}^{2\times \frac{Iteration}{Max\left(Iter\right)}} \end{array}$$ (23)

The behavior of an algorithm can be greatly impacted by the creation of eddies or Fish Aggregation Devices (FADs). These phenomena act as operators that prevent the algorithm from being stuck in local maxima, which affects the overall optimization process. Longer hops are used in order to overcome local optima stagnation during the simulation. The effect of FADs is outlined below by the Equation 24:


$$\begin{array}{l} Prey_{k}=\{\begin{array}{c} Prey_{k}+CF\left[(S_{\max}-\text{S})⊗M+S_{\min}\right]⊗U,if,r\leq FADs\\ Prey_{k}+(Prey_{k}-Prey_{k})\left[(1-r)FADs+r\right],if,r>FADs \end{array} \end{array}$$ (24)

The stages and adaptive approaches outlined describe the dynamics of the Marine Predators Algorithm. These strategies enable it to explore the optimization domain successfully while tackling various scenarios and shortcomings. Algorithm 2 illustrates the operational process of QMPA.

Algorithm 2 Working mechanism of QMPA.

1 Initialize the population size N and
  Max_Iteration
2 Set the initial fitness values for the
  optimal solution *Prey*_*k*_ and the global best
  solution *Elite*_*k*_.
3 While (t < Max_Iteration )
4     Upgrade *Prey*_*k*_ and *Elite*_*k*_,
5     Compute self-adaptive weight ω
6     for k = 1 to N
7     if t < Max_Iteration/3
8       Update the present position *Prey*_*k*_
      utilizing Equation 20
9     if t >
    Max_Iteration/3 & t < 2^*^ Max_Iteration/3
10       Update the present position *Prey*_*k*_
      utilizing Equation 22
11     if t > 2^*^ Max_Iteration/3
12        Update the present position *Prey*_*k*_
       utilizing Equation 23
13 End for
14 If (f(*Prey*_*k*_) < f (*Elite*_*k*_))
15       Update the optimal location *Elite*_*k*_ and
      *Prey*_*k*_
16 End if
17       Apply the FADs effect to update *Prey*_*k*_.
18 End while
19 Return *Elite*_*k*_

### 3.7 Deep extreme neural networks

This kind of neural network employs a single hidden layer, where the layer does not necessarily need tuning. Compared to the various ML approaches like SVM and Random Forest (RF), the Extreme Learning Machine (ELM) shows better efficiency, high speed, and reduced processing overhead. ELM utilizes kernel-based transformations to provide high predictive accuracy and enhanced overall performance. Its key advantages are low training error and a high level of approximation, largely due to ELM being able to automatically optimize its weight parameters and using effective, non-zero activation functions. The complete workflow of ELM is described in Wang et al. (30). The input features to ELM (integrating the spatial and temporal features) are shown in Equation 25


$$\begin{array}{l} X\left(n\right)=\overset{L}{\underset{i=0}{\sum }}F\left(Fused\left(i\right)\right) \end{array}$$ (25)

Here, *Y* signifies the fused features of the network shown in Equation 26.

ELM output formulation is as follows:


$$\begin{array}{l} Y\left(n\right)=\;X\left(n\right)\beta =X\left(n\right)\;X^{T}{\left(\frac{1}{C}XX^{T}\right)}^{-1}O \end{array}$$ (26)

To distinguish the outputs effectively, the binary cross-entropy method is employed to calculate the loss function, and its mathematical model is given as shown in Equation 27


$$\begin{array}{l} Loss=(\frac{1}{K})\overset{K}{\underset{i=1}{\sum }}(Y(i)*Log\;Y^{\prime })+\eta ||\theta {||}^{2} \end{array}$$ (27)

Here, *K* represents the dimension of the capsule feature vectors, η represents a regularization parameter, and |θ| is referred to as a constant.

To reduce further complexity, hyperparameter optimization is carried out to identify the optimal network settings, thus reducing the complexity of the model.

### 3.8 QMPA tuned DFF classification network

Hyperparameter optimization is utilized to determine the most appropriate parameters for the suggested framework. This process is conducted prior to model training. The hyperparameters considered for tuning in this research involve the hidden layer counts, dropout rate, number of epochs, batch size, and the number of hidden units. Hyperparameter optimization methods are utilized to fine-tune these network parameters, thereby enhancing the efficiency of the classification procedure.

The dense layer's weights in the DENN classifier are optimized using QMA. Initially, hyperparameters are randomly sampled at the start of training. The fitness of the proposed method is assessed according to Equation 28, and tuning parameters are iteratively computed using Algorithm 2. Each iteration concludes once the FF satisfies the criteria defined in Equation 28.


$$\begin{array}{l} FF=\;Maximum\left\{(1-Accuracy\right)+(1-Precision)\\ \;+\left(1-Recall)\right\} \end{array}$$ (28)

### 3.9 LLM layers

**Figure 7:**
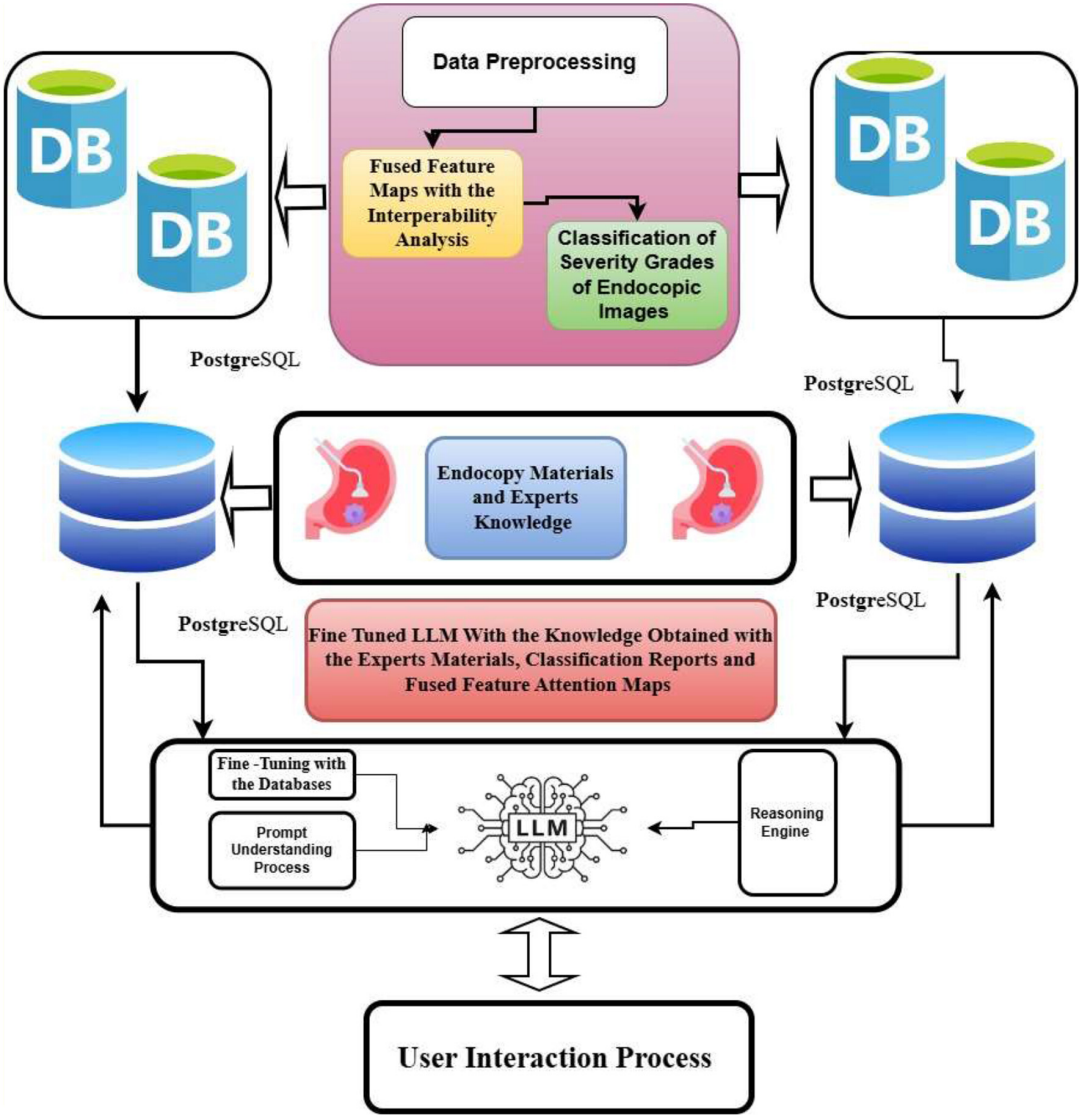
Suggested LLM model integrated for the proposed framework for the better explainability.

Finally, the LLM layers are trained with the classification reports obtained from the proposed layers and medical prompts so that the users can interact with the hospitals with an accurate diagnosis of the patients. The generated feature maps and classification reports in accordance with abnormalities and vascular patterns, along with the textual irregularities, are often incorporated as clinical reasoning features for the LLMs. To optimize the interpretability and clinical reliability of automated endoscopic image analysis systems, we integrate a Large Language Model (LLM) with the proposed multi-graded attention fusion framework. The multi-graded attention module produces hierarchical attention responses from the convolutional encoder at four semantic depths (i.e., low-level texture, mid-level structural, high-level semantic, and deep clinical layers). These attention maps, generated using the suggested framework, highlight discriminative regions corresponding to mucosal abnormalities, polyp boundaries, vascular patterns, and textural irregularities in the Kvasir dataset. In the first phase, the hierarchical layered fusion feature maps are encoded into a structured visual-token representation, which is then offered as contextual input to the LLM. Subsequently, the LLM interprets spatial activation patterns and correlates them with domain-specific endoscopic knowledge, thereby generating human-readable diagnostic explanations. Such explanations include references to abnormal mucosal regions, lesion boundaries, morphological deviations, or color/textural anomalies, which are crucial for clinical decision-making. Moreover, the LLM automatically generates structured clinical reports from fused attention responses. By combining the attention-informed visual reasoning with diagnosis reports, the LLM produces high-level summaries, descriptive lesion characterizations, and potential differential diagnoses. This multimodal integration bridges the gap between computational feature extraction and clinical interpretation, offering a transparent, explainable, and clinician-aligned computer-aided diagnosis pipeline. Figure 7 presents the layers of LLM used in the research. The research incorporates the GPT-4o as the main LLM core interfaced with PostgreSQL for storing the vector images and documents, which are used as the main training and fine-tuning procedure.

## 4 Experimental implementation and validation process

This section discusses the implementation details, performance evaluation, and result outcomes with the evaluation metrics.

### 4.1 Implementation details

The suggested framework was fully executed utilizing the Python (3.12) environment, which includes essential libraries such as Plotly for data representation, Pandas for data handling, and NumPy and Seaborn for numerical processing and statistical visualization. PyTorch serves as the primary framework for implementing the suggested algorithm. The experimental configuration was implemented on a high-performance computing workstation equipped with an Intel Core i9 processor, an NVIDIA Tesla 1,024 GB GPU to accelerate computational tasks, and 32 GB of memory operating at 3.4 GHz. Table 2 summarizes the optimized training parameters used for deploying the proposed model.

**Table 2:** Optimized hyperparameters utilized for configuring the suggested framework.

S. No	Hyperparameters	Details
01	Initial learning rate	0.001
02	Momentum	0.021
03	Optimizer	ADAM
04	Batch Size	40
05	Epochs Count	240

### 4.2 Evaluation metrics

The evaluation of the suggested research is analyzed utilizing classification metrics. The classification metrics, such as accuracy, precision, recall, specificity, and F1-score, are used to assess the effectiveness of the model in classifying the various levels of gastric cancers. Table 3 illustrates the mathematical formulation for computing the classification measures.

**Table 3:** Mathematical expression for calculating the classification metrics.

S. No	Performance metrics	Expression
01	Accuracy	$\frac{TP+TN}{TP+TN+FP+FN}$
02	Recall	$\frac{TP}{TP+FN}$ × 100
03	Precision	$\frac{TP}{TP+FP}$
04	F1-Score	$2.\frac{Precison\times Recall}{Precision+Recall}$

### 4.3 Assessment analysis

The efficiency of the suggested algorithm was examined utilizing the performance measures mentioned in Table 3. The evaluation metrics were utilized to compare the suggested methodology with the SOTA model current procedures in emphasize to highlight the key strengths of our approach. The confusion matrix and receiver operating characteristics (ROC-AUC) are used for evaluating the suggested model.

**Figure 8:**
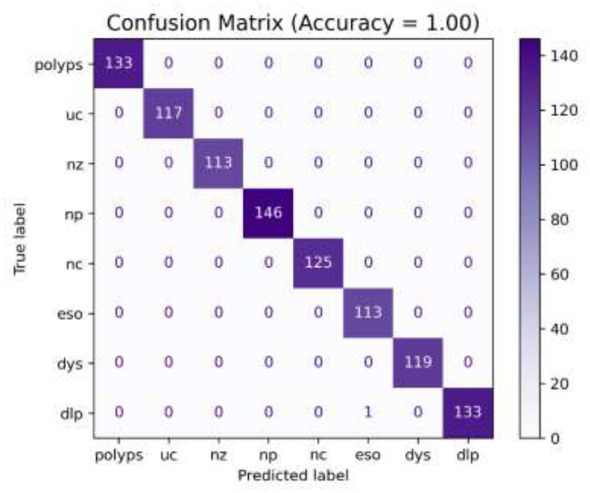
Confusion matrix of the recommended methodology (using the fused features) for detecting the severity Grade-0.

**Figure 9:**
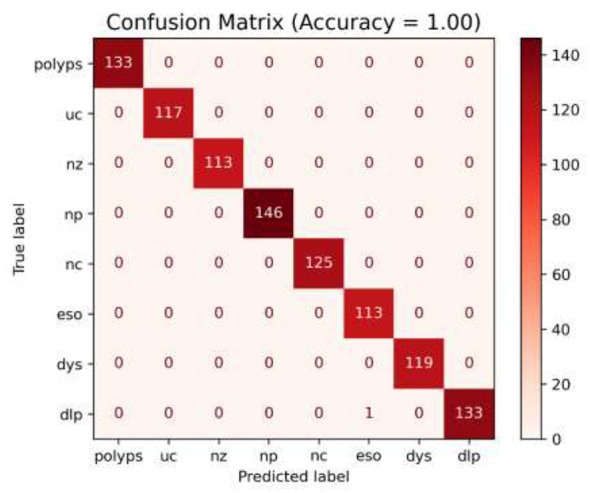
Confusion matrix for the suggested framework (utilizing the fused features) for detecting the severity Grade-1 and Grade-2.

**Figure 10:**
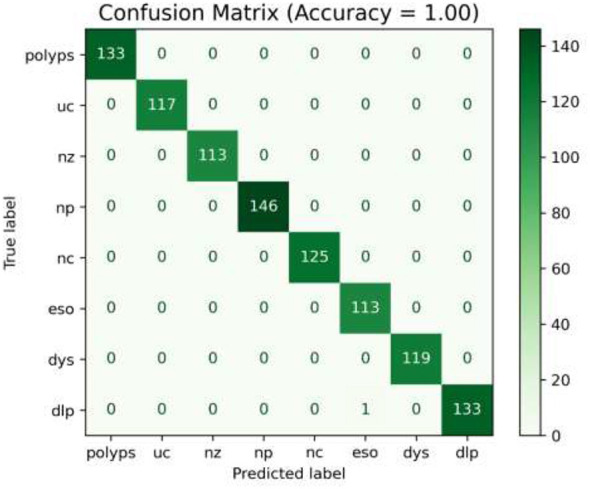
Confusion matrix of the recommended methodology (using the fused features) for detecting the severity Grade-3.

**Figure 11:**
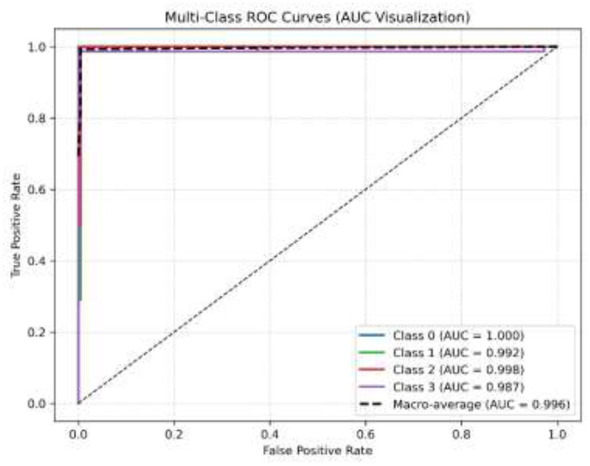
Average AUC-ROC of the suggested algorithm (using the fused features) for detecting the different grades.

**Figure 12:**
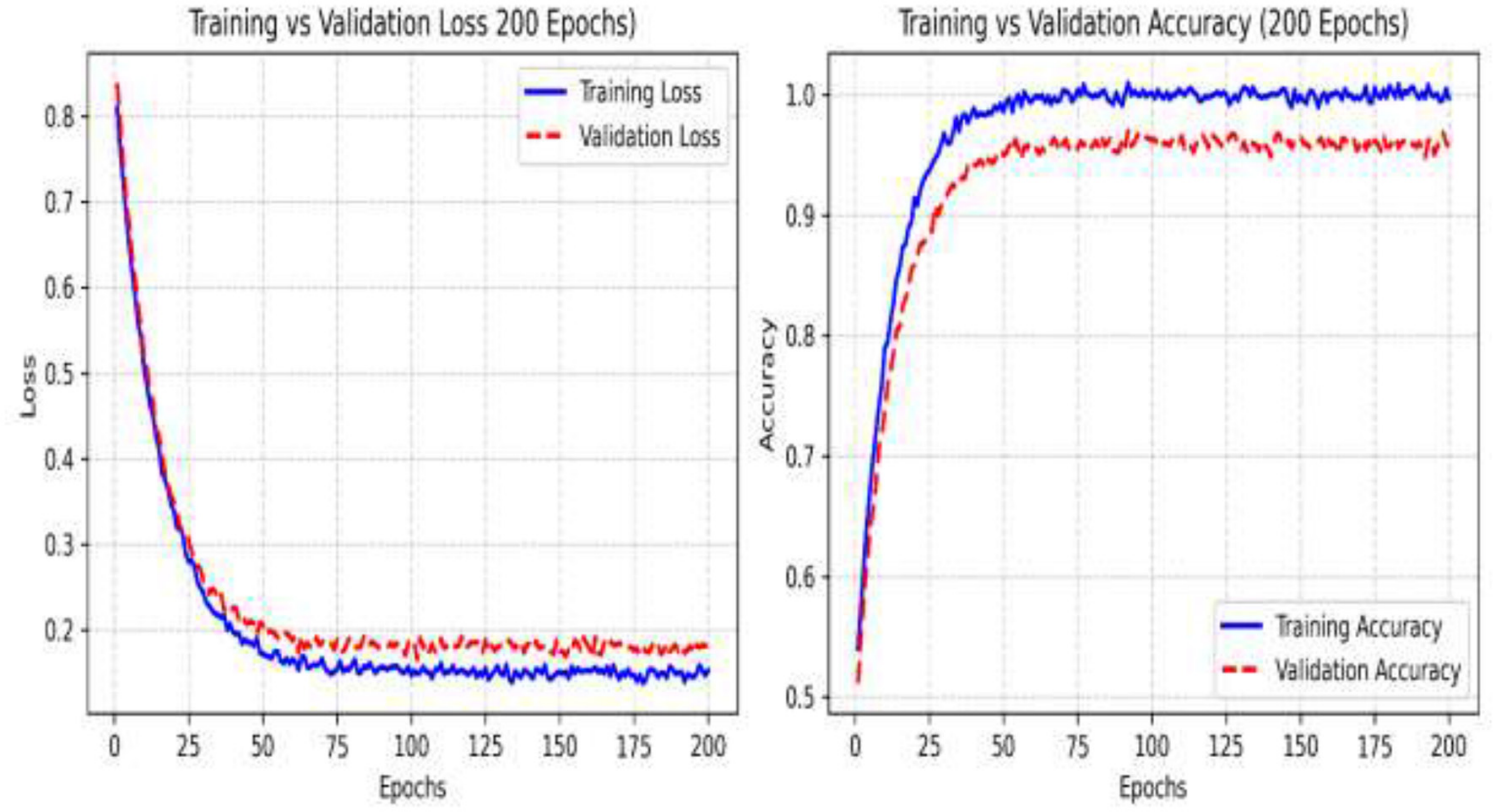
Training and validation performance of the recommended algorithm in recognizing the different severity grades.

In the initial phase of experimentation, the effectiveness of the suggested approach was examined utilizing the confusion matrix along with ROC-AUC in detecting the severity grades of 8 classes of GCs. Figures 8–10 demonstrate the confusion matrix of the suggested approach in detecting the severity grades of GCs. The confusion matrix of the suggested approach has demonstrated the excellence of the model in producing the maximum accuracy of 1.00 in differentiating the severity grades in the endoscopic images. The results can show the model effectively minimizes the positive and negative rates while accurately detecting the overall results. This has been proved in Figure 11, in which the average ROC-AUC of the suggested model has maintained the accuracy of 0.999 in detecting the severity grades in 8 classes of GCs. The model has detected both positive and negative samples properly, and the algorithm strikes a balance between the precision and recall by ensuring a high F1-score, which plays a major role in the classification of malignant grades in the context of gastrointestinal cancers. Figure 12 demonstrates the loss and validation performance of the model with the increased number of epochs. These plots provide the process of optimization and the generalization process of the model beyond the training datasets. From Figure 12, it is very clear that the difference in the two values of loss is very low, which prevents the sentiments of overfitting and poor robustness. Tables 4–6 illustrate the classification efficiency of the algorithm in recognizing the severity grades for the individual classes.

**Table 4:** Performance of the proposed algorithm in recognizing the individual classes with the severity grades (Grade 0).

Classes of detection	Evaluation measures			
	Accuracy	Precision	Recall	F1-score
Ulcerative-colitis	1.00	0.999	0.999	1.00
Polyps	0.998	0.992	0.991	1.00
Normal-z-line	0.999	0.993	0.992	0.99
Normal-pylorus	1.00	0.992	0.992	0.99
Normal-cecum	0.9993	0.9992	0.9990	0.995
Esophagitis	0.9993	0.9994	0.993	0.9934
Dyed-resection-margins	0.9990	0.9989	0.9988	0.9988
Dyed-lifted-polyps	0.9998	0.9995	0.9994	0.998

Tables 4–6 present the average performance of the suggested methodology in classifying the various severity grades of individual classes. The recommended algorithm has produced the average performance of reaching the accuracy of 0.999, precision of 0.998, recall of 0.997, and F1-score of 0.99 in differentiating the different severity grades of the individual classes in GCs. Table 4 presents the algorithm's ability in detecting the Grade-0 for individual classes. From Tables 5, 6, the suggested algorithm has yielded uniform performance in detecting the different classes of severity grades by attaining the accuracy, precision, recall, and F1-score values of 0.998, 0.997, 0.997, and 1.00, respectively. The integration of the MHDRAM in the Swin V2 Model and QMA optimized classification networks with the fused features has achieved the highest performance in classifying the different severity classes by the types of GCs.

**Table 5:** Performance of the proposed algorithm in recognizing the individual classes with the severity grades (Grade 1 and Grade 2).

Classes of detection	Evaluation measures			
	Accuracy	Precision	Recall	F1-score
Ulcerative-colitis	1.00	0.999	0.999	1.00
Polyps	0.998	0.992	0.991	1.00
Normal-z-line	0.999	0.993	0.992	0.99
Normal-pylorus	1.00	0.992	0.992	0.99
Normal-cecum	0.9993	0.9992	0.9990	0.995
Esophagitis	0.9993	0.9994	0.993	0.9934
Dyed-resection-margins	0.9990	0.9989	0.9988	0.9988
Dyed-lifted-polyps	0.9998	0.9995	0.9994	0.998

**Table 6:** Performance of the recommended approach in detecting the individual classes with the severity grades (Grade 3).

Classes of detection	Evaluation measures			
	Accuracy	Precision	Recall	F1-score
Ulcerative-colitis	1.00	0.999	0.999	1.00
Polyps	0.998	0.992	0.991	1.00
Normal-z-line	0.999	0.993	0.992	0.99
Normal-pylorus	1.00	0.992	0.992	0.99
Normal-cecum	0.9993	0.9992	0.9990	0.995
Esophagitis	0.9993	0.9994	0.993	0.9934
Dyed-resection-margins	0.9990	0.9989	0.9988	0.9988
Dyed-lifted-polyps	0.9998	0.9995	0.9994	0.998

### 4.4 Comparative analysis with existing models

To validate the ability of the suggested methodology compared to other existing systems, existing frameworks (31–34) are deployed for the experimentation process with the same datasets that are used for evaluating and validating the suggested model.

**Figure 13:**
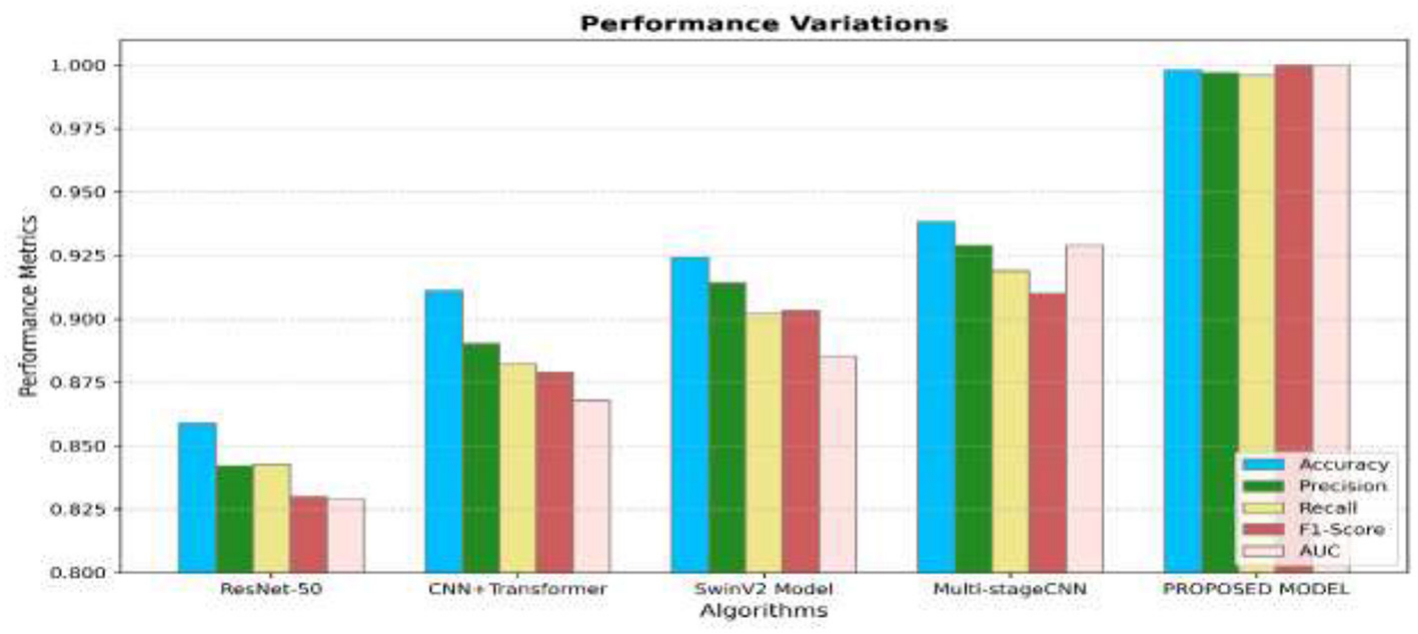
Average performance of the distinct algorithms in detecting the severity grades of 8 classes using only endoscopic features.

**Figure 14:**
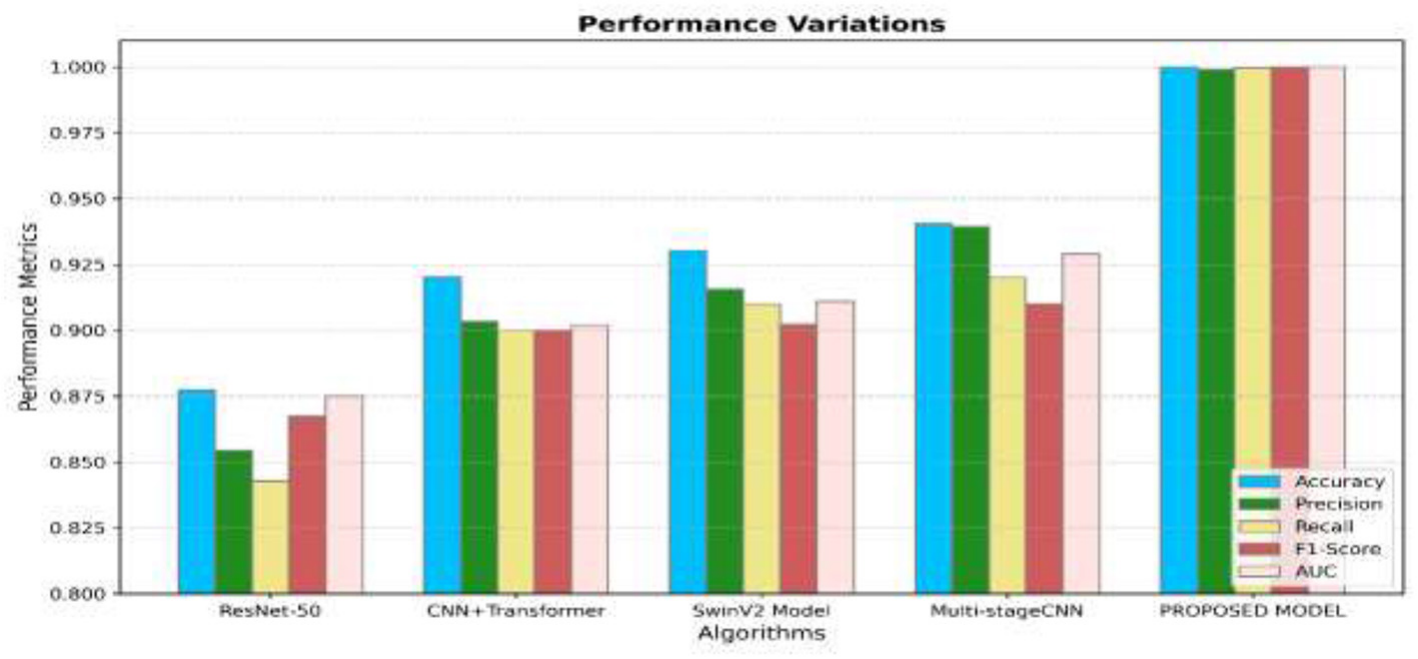
Average performance of the distinct algorithms in recognizing the severity grades of 8 classes using fused features.

Figure 13 illustrates the efficiency of the different algorithms in recognizing the severity grades of 8 classes with only endoscopic features. From Figure 13, the model produces the highest performance by attaining the accuracy, precision, recall, and F1-score values of 0.998, 0.997, 0.9, and 0.99, respectively, and outperforms the other existing models. The existing Multi-stage CNN has considerable performance in detecting the various grades of the GC classes. Figure 14 illustrates that the efficiency of all algorithms has been enhanced by 5% in detecting the different grades of GCs. Though the performance has been improved, the suggested approach has produced high performance by obtaining the accuracy, precision, recall, and F1-score values of 1.00, 0.999, 0.9, and 1.00, respectively. From Figures 13, 14, it is apparent that the suggested approach has surpassed the multi-stage CNN by 10.7%, the SwinV2 Model by 15%, and other models by 20% in classifying the different lesions of GC. From the analysis, it has been concluded that the suggested approach has attained the most promising performance in recognizing the different severity grades in the GC classes.

### 4.5 Interpretability analysis

**Figure 15:**
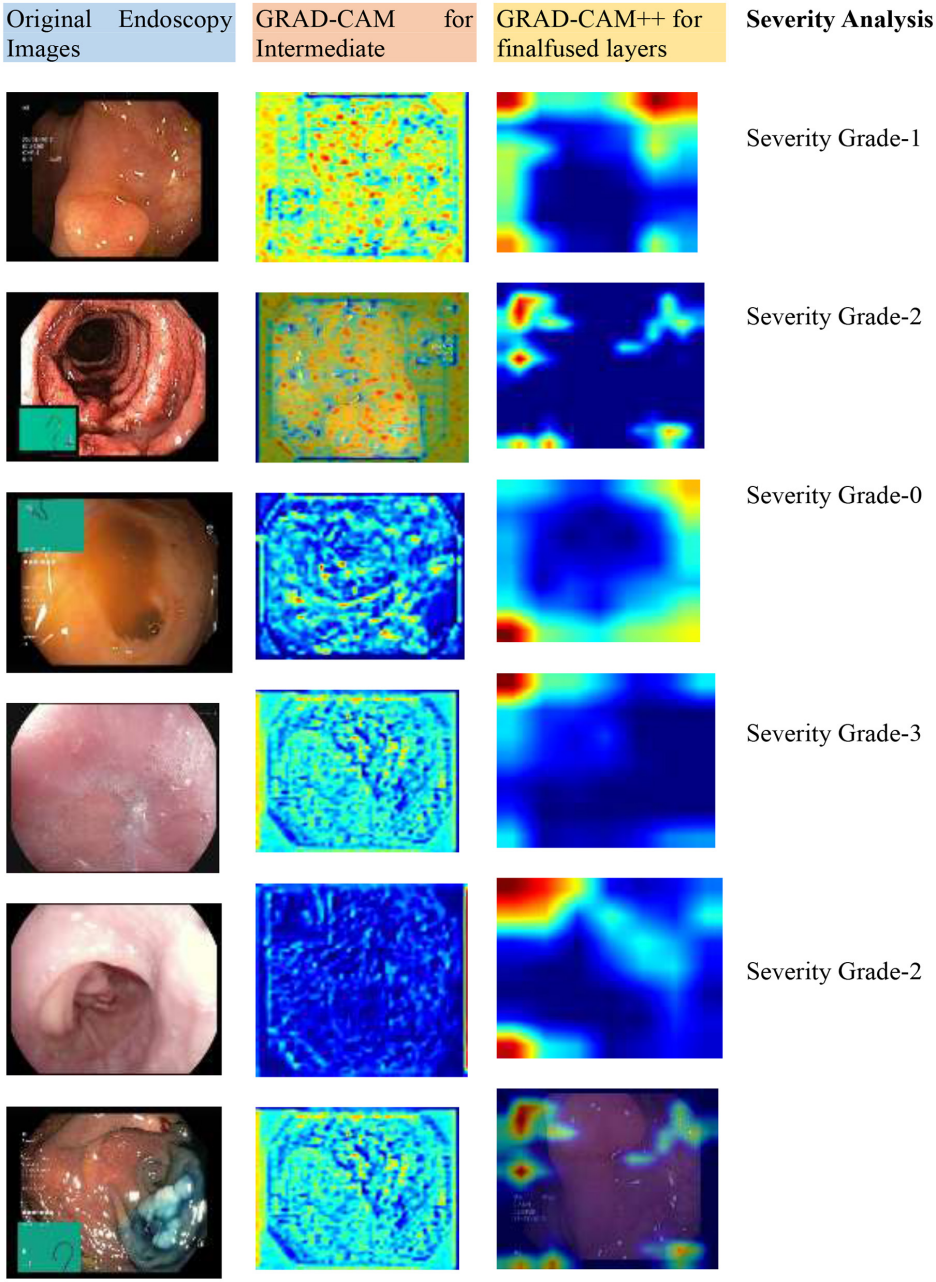
GRAD-CAMM analysis for the input endoscopic images.

Figure 15 illustrates that the model is able to identify symptoms of a disease effectively while providing a clear view of the spatial characteristics identified by the model, thus making the disease diagnosis process more interpretable. Figure 15 shows the Grad-CAM analysis highlighting the discriminative regions activated by the model for the input endoscopic images. In short, the results obtained substantiate the robustness, scalability, and explainability of the heterogeneous transformer-based ensemble model.

### 4.6 LLM clinical decision analysis

**Figure 16:**
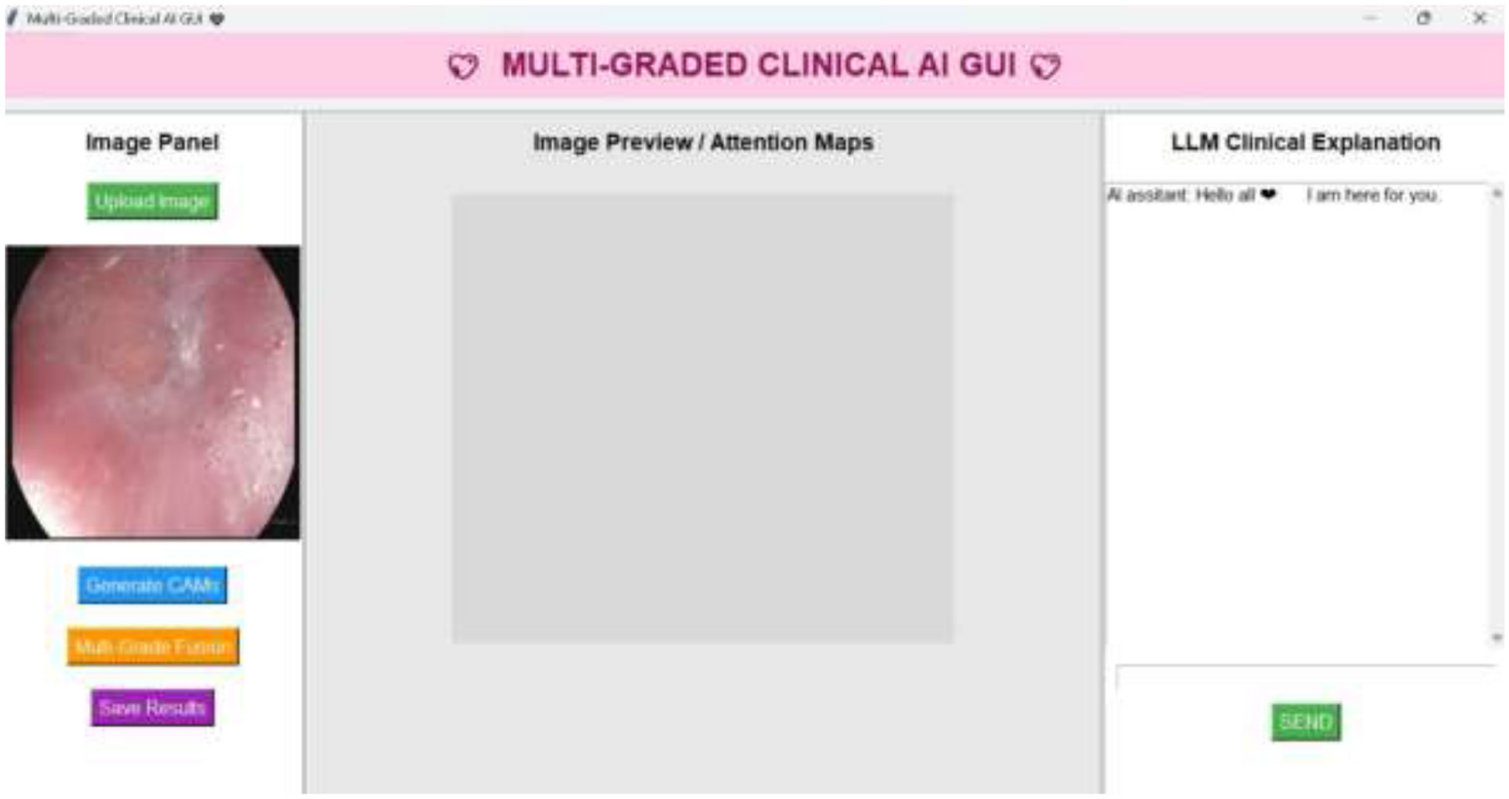
LLM-based user interaction designed for an effective interpretability analysis.

Figure 16 presents the LLM-based interactive user interfaces with the interoperability and classification results with the GPT4.0 using the OpenAI API keys. The clinical explanations are evaluated using LLM scores such as the BLEU score, ChRF, METEOR, and expert clinical analysis. The performance of the various LLM models (35–40) has been evaluated using the metrics mentioned, and it is presented in Table 7.

**Table 7:** Different LLM performances in terms of producing more meaningful insights for clinical decisions.

LLM models	Performance metrics				
	BLEU	chRF	WER	METEOR	ECS
GEMMA	0.84	0.75	0.19	0.84	0.8
LLAMA	0.87	0.743	0.184	0.87	0.8
GEMINI	0.86	0.732	0.192	0.86	0.8
MedGROK	0.87	0.750	0.188	0.87	0.8
GPT3.0	0.86	0.75	0.187	0.86	0.8
GPT3.5	0.84	0.73	0.178	0.84	0.86
GPT4.0 (Go)	0.91	0.788	0.177	0.91	0.88

The response from the LLM model has been evaluated using the 10 clinical experts, and performance is calculated and presented in Table 7. From Table 7, it is evident that all the LLM models have increased the interpretability strength of the deep learning model. From the analysis, GPT4.0 has surpassed the other LLMs in terms of attaining the maximum BLEU scores and finds its place in increasing the clinical decisions and support management systems. From the experimental analysis, the performance of any Explainable AI techniques can be enhanced by integrating with the LLM models.

### 4.7 Statistical outcomes

Following the PET analysis in binary classification outcomes, the findings of the proposed approach have been statistically validated. The comprehensive assessment of distinct algorithms is carried out by evaluating their unique strengths and inherent shortcomings. Table 8 provides the statistical verification of multiple meta-heuristic algorithms combined with the recommended model, in which the derived results are produced and benchmarked against the suggested framework. The nature-inspired algorithms utilized for execution are Particle Swarm Optimization (PSO), Spider Optimization Algorithm (SOA), Whale Optimization Algorithm (WOA), genetic algorithm (GA), Sand cat optimization (SCO), and White Shark Optimizers (WSO). These optimizers are included in the suggested DL frameworks, and validation is carried out by comparing with the Quantum Marine Predator Algorithm in the model. Conversely, the quantitative results, derived from the FF assessment across 100 iterations, are presented as the best, worst, mean, median, standard deviation (SD), variance, and confidence interval (CI).

**Table 8:** Fitness function-driven results for the distinct combinations of optimizers.

Algorithm	Best	Worst	Mean	Median	SD	Variance	CI
PSO	0.7490	0.6435	0.72010	0.022819	0.06530	7.42 × 10^−6^	0.552, 0.888
GA	0.730333	0.63527	0.69036	0.020204	0.07035	6.392 × 10^−6^	0.509, 0.871
WOA	0.7525	0.6767	0.65376	0.027825	0.068907	5.897 × 10^−5^	0.476, 0.831
SOA	0.77437	0.6903	0.71242	0.02043	0.054639	4.1293 × 10^−4^	0.571, 0.853
SCO	0.80236	0.64391	0.73405	0.030206	0.059035	3.905 × 10^−4^	0.582, 0.886
WSO	0.85637	0.69036	0.74024	0.039305	0.045892	3.6792 × 10^−4^	0.622, 0.858
Proposed algorithm	0.99764	0.81203	0.85641	0.06345	0.037631	2.28931 × 10^−4^	0.759, 0.953

Table 8 illustrates the performance outcomes obtained from various optimizer combinations applied to the suggested network architectures. From Table 8, it is clearly observed that the recommended algorithm delivers superior results compared with all other optimization techniques. The model's stability is also evident in Table 8, consistently demonstrating stronger and more reliable performance than the existing algorithms.

### 4.8 Ablation experiments

The ablation study validates the cumulative effect of each component in the proposed system. The baseline model (SwinV2 + ELM) reported an accuracy of 0.892, which was moderate in terms of discriminative power. The addition of MHDRAM enhanced the accuracy to 0.918, which was effective in dealing with multi-scale contextual feature extraction, and the addition of Sparse GRU further enhanced the accuracy to 0.936, which was efficient in dealing with sequential patterns. The addition of Cross-Attention Fusion (MSMCAM) further boosted the accuracy to 0.954, which emphasized the effectiveness of multimodal feature fusion. With the use of QMPA-based optimization, the accuracy was further enhanced to 0.972, which proved the efficiency of convergence and parameter optimization. Finally, the overall model reported an accuracy of 0.99, precision of 0.997, recall of 0.99, and F1-score of 0.99, which validated the efficacy of the proposed integrated framework for gastrointestinal severity prediction (Table 9).

**Table 9:** Ablation study for the suggested system.

Model configuration	SwinV2	MHDRAM	Sparse GRU	MSMCAM	QMPA	ELM	Accuracy	Precision	Recall	F1-score
Baseline (SwinV2 + ELM)	✓	✗	✗	✗	✗	✓	0.892	0.884	0.879	0.881
+ MHDRAM	✓	✓	✗	✗	✗	✓	0.918	0.910	0.905	0.907
+ Sparse GRU	✓	✓	✓	✗	✗	✓	0.936	0.929	0.924	0.926
+ Cross-attention fusion (MSMCAM)	✓	✓	✓	✓	✗	✓	0.954	0.947	0.943	0.945
+ QMPA optimization	✓	✓	✓	✓	✓	✓	0.972	0.968	0.965	0.966
Proposed model	✓	✓	✓	✓	✓	✓	0.99	0.997	0.99	0.99

## 5 Conclusion and future enhancement

This research introduces a new LLM-integrated Explainable Swin architecture for an accurate classification of gastric cancers utilizing endoscopic and CT scan images. The proposed framework introduces multi-headed dilated residual attention networks in the Swinv2 Model and Sparsity in the GRU networks for an effective feature extraction process. The novel encoder–decoder architecture with the Modified SwinV2 and Sparse GRU Networks is formulated to extract the multi-scale features from both CT and endoscopic images. Both features are fused by the Modal Cross-attention layers, which are used to train the Quantum marine Evoked Classification networks for an effective detection of severity grades in GCs. Finally, GPT4.0-based LLM has been integrated to increase the interpretability of the decision process. The comprehensive assessment has been executed utilizing Kvasir and TCIA CT images, in which their evaluation metrics are measured and benchmarked against other DL frameworks. Experimental results prove that the fused features with the hybrid learning models have produced the peak performance, such as an accuracy of 0.99, precision of 0.997, recall of 0.99, and F1-score of 0.99, respectively. Furthermore, the response of the LLM model with the GRAD-CAM technique has been analyzed by clinical experts, and its performance was found to be better than the traditional interpretability methods. As a future improvement, the computational complexity will be minimized to facilitate its execution on devices with limited resources. The LLM models will be fine-tuned to support multilingual clinical communication. In addition, the proposed framework will be extended toward real-time clinical application through prospective multi-center data collection and validation studies to enhance its practical applicability. Furthermore, future research will focus on addressing practical challenges associated with integrating the proposed framework into existing hospital diagnostic workflows, including ensuring compatibility with Hospital Information Systems (HISs) and PACS infrastructure, maintaining data privacy and regulatory compliance, minimizing workflow disruption, and strengthening clinician trust through transparent AI-assisted decision support mechanisms.

## Data Availability

The original contributions presented in the study are included in the article/supplementary material, further inquiries can be directed to the corresponding authors.

## References

[1] Arnold M Abnet CC Neale RE Vignat J Giovannucci EL McGlynn KA . Global burden of 5 major types of gastrointestinal cancer. Gastroenterology. (2020) 159:335–49. doi: 10.1053/j.gastro.2020.02.068 32247694 PMC8630546

[2] Ferlay J Ervik M Lam F Colombet M Mery L Piñeros M . Global Cancer Observatory: Cancer Today. Lyon: International Agency for Research on Cancer (2020).

[3] Huang J Lok V Ngai CH Zhang L Yuan J Lao XQ . Worldwide burden of, risk factors for, and trends in pancreatic cancer. Gastroenterology. (2021) 160:744–54. doi: 10.1053/j.gastro.2020.10.007 33058868

[4] Li N Wu P Shen Y Yang C Zhang L Chen Y . Predictions of mortality related to four major cancers in China, 2020 to 2030. Cancer Commun. (2021) 41:404–13. doi: 10.1002/cac2.12143 33660417 PMC8118592

[5] Al-Ishaq RK Overy AJ Büsselberg D. Phytochemicals and gastrointestinal cancer: cellular mechanisms and effects to change cancer progression. Biomolecules. (2020) 10:105. doi: 10.3390/biom10010105 31936288 PMC7022462

[6] Luan S Yu X Lei S Ma C Wang X Xue X . Deep learning for fast super-resolution ultrasound microvessel imaging. Phys Med Biol. (2023) 68:245023. doi: 10.1088/1361-6560/ad0a5a 37934040

[7] Yu X Luan S Lei S Huang J Liu Z Xue X . Deep learning for fast denoising filtering in ultrasound localization microscopy. Phys Med Biol. (2023) 68:205002. doi: 10.1088/1361-6560/acf98f 37703894

[8] Song W Wang X Guo Y Li S Xia B Hao A. CenterFormer: a novel cluster center enhanced transformer for unconstrained dental plaque segmentation. IEEE Trans Multimed. (2024) 26:10965–78. doi: 10.1109/TMM.2024.3428349

[9] Xu X Luo Q Wang J Song Y Ye H Zhang X . Large-field objective lens for multi-wavelength microscopy at mesoscale and submicron resolution. Opto-Electron Adv. (2024) 7:230212. doi: 10.29026/oea.2024.230212

[10] Zhou J Guo Z Peng X Wu B Meng Q Lu X . Chrysotoxine regulates ferroptosis and the PI3K/AKT/mTOR pathway to prevent cervical cancer. J Ethnopharmacol. (2025) 338:119126. doi: 10.1016/j.jep.2024.119126 39557107

[11] Zheng Q Zheng H Liu Z Guo W Li S Ma J . Current status and future potential of radiomics in the management of patients with gastric cancer. Adv Radiother Nucl Med. (2025) 3:24–38. doi: 10.36922/arnm.8350

[12] Jiang R Yin X Yang P Cheng L Hu J Yang J . A transformer-based weakly supervised computational pathology method for clinical-grade diagnosis and molecular marker discovery of gliomas. Nat Mach Intell. (2024) 6:876–91. doi: 10.1038/s42256-024-00868-w

[13] Ye Z Zhang Y Liang Y Lang J Zhang X Zang G . Cervical cancer metastasis and recurrence risk prediction based on deep convolutional neural network. Curr Bioinform. (2022) 17:164–73. doi: 10.2174/1574893616666210708143556

[14] Xu WX Qu Q Zhuang HH Teng XQ Wei YW Luo J . The burgeoning significance of liquid-liquid phase separation in the pathogenesis and therapeutics of cancers. Int J Biol Sci. (2024) 20:1652–68. doi: 10.7150/ijbs.92988 38481812 PMC10929184

[15] Saxena S Rathor S. An ensemble-based model of detecting plant disease using CNN and random forest. In: 2023 6th International Conference on Information Systems and Computer Networks (ISCON). Mathura: IEEE (2023). p. 1–6. doi: 10.1109/ISCON57294.2023.10112023

[16] Ma J Yang F Yang R Li Y Chen Y. Interpretable deep learning for gastric cancer detection: a fusion of AI architectures and explainability analysis. Front Immunol. (2025) 16:1596085. doi: 10.3389/fimmu.2025.1596085 40510366 PMC12159836

[17] Huang ZN Zhang HX Sun YQ Zhang XQ Lin YF Weng CM . Multi-cohort study in gastric cancer to develop CT-based radiomic models to predict pathological response to neoadjuvant immunotherapy. J Transl Med. (2025) 23:362. doi: 10.1186/s12967-025-06363-z 40128827 PMC11934467

[18] Yao Y Cen X Gan L Jiang J Wang M Xu Y . Automated esophageal cancer staging from free-text radiology reports: large language model evaluation study. JMIR Med Inform. (2025) 13:e75556. doi: 10.2196/75556 41105871 PMC12533932

[19] Wan XH Liu MX Zhang Y Kou GJ Xu LQ Liu H . DeepGut: a collaborative multimodal large language model framework for digestive disease assisted diagnosis and treatment. World J Gastroenterol. (2025) 31:109948. doi: 10.3748/wjg.v31.i31.109948 40901691 PMC12400200

[20] Khayatian D Maleki A Nasiri H Dorrigiv M. Histopathology image analysis for gastric cancer detection: a hybrid deep learning and catboost approach. Multimed Tools Appl. (2025) 84:21777–803. doi: 10.1007/s11042-024-19816-2

[21] Zubair M Owais M Hassan T Bendechache M Hussain M Hussain I . An interpretable framework for gastric cancer classification using multi-channel attention mechanisms and transfer learning approach on histopathology images. Sci Rep. (2025) 15:13087. doi: 10.1038/s41598-025-97256-0 40240457 PMC12003787

[22] Zhang R-Y Qiang P-P Hao Y-X Tan H-Y Zhao K Cai L-J . GutGPT: a multidimensional knowledge-enhanced large language model for gastrointestinal medicine. J Biomed Inform. (2025) 169:104885. doi: 10.1016/j.jbi.2025.104885 40720988

[23] Qin Y Chang J Li L Wu M. Enhancing gastroenterology with multimodal learning: The role of large language model chatbots in digestive endoscopy. Front Med. (2025) 12:1583514. doi: 10.3389/fmed.2025.1583514 40470039 PMC12133735

[24] Binzagr F. Explainable AI-driven model for gastrointestinal cancer classification. Front Med. (2024) 11:1349373. doi: 10.3389/fmed.2024.1349373 38686367 PMC11056557

[25] Mudavadkar GR Deng M Al-Heejawi SMA Arora IH Breggia A Ahmad B . Gastric cancer detection with ensemble learning on digital pathology: use case of gastric cancer on GasHisSDB dataset. Diagnostics. (2024) 14:1746. doi: 10.3390/diagnostics14161746 39202233 PMC11354078

[26] Kaggle. Available online at: https://www.kaggle.com/datasets/yasserhessein/the-kvasir-dataset (Accessed December, 2025).

[27] Karras T Laine S Aittala M Hellsten J Lehtinen J Aila T. Analyzing and improving the image quality of StyleGAN. In: 2020 IEEE/CVF Conference on Computer Vision and Pattern Recognition (CVPR). Seattle, WA: IEEE (2020). p. 8107–16. doi: 10.1109/CVPR42600.2020.00813

[28] Chung J Gulcehre C Cho K Bengio Y. Empirical evaluation of gated recurrent neural networks on sequence modeling. arXiv [Preprint]. (2014) arXiv:1412.3555. Available online at: https://arxiv.org/abs/1412.3555 (Accessed December, 2025).

[29] Nouman Noor M Nazir M Khan SA Ashraf I Song O-Y. Localization and classification of gastrointestinal tract disorders using explainable AI from endoscopic images. Appl Sci. (2023) 13:9031. doi: 10.3390/app13159031

[30] Wang J Lu S Wang SH Zhang Y. A review on extreme learning machine. Multimed Tools Appl. (2022) 81:41611–60. doi: 10.1007/s11042-021-11007-7

[31] Cambay VY Barua PD Hafeez Baig A Dogan S Baygin M Tuncer T . Automated detection of gastrointestinal diseases using Resnet50^*^-based explainable deep feature engineering model with endoscopy images. Sensors. (2024) 24:7710. doi: 10.3390/s24237710 39686247 PMC11644848

[32] Fu B Zhang M He J Cao Y Guo Y Wang R. StoHisNet: a hybrid multi-classification model with CNN and transformer for gastric pathology images. Comput Methods Programs Biomed. (2022) 221:106924. doi: 10.1016/j.cmpb.2022.106924 35671603

[33] Fahad M Mobeen NE Shariq AI Cheikh FA Daudpota SM Ullah M. Advancing gastrointestinal disease diagnosis: a fine-grained approach using swin transformer and explainable AI techniques. In: 2024 12th European Workshop on Visual Information Processing (EUVIP). Geneva: IEEE (2024). p. 1–6. doi: 10.1109/EUVIP61797.2024.10772988

[34] Dugăeşescu A Chiru C-M Nan M Trăscău M Florea AM. Explainable cancer segmentation through classification. In: 2024 IEEE 20th International Conference on Intelligent Computer Communication and Processing (ICCP). Cluj-Napoca: IEEE (2024). p. 1–8. doi: 10.1109/ICCP63557.2024.10793024

[35] Gao M Lu P Zhao Z Bi X Wang F. Leveraging large language models: enhancing retrieval-augmented generation with ScaNN and gemma for superior AI response. In: 2024 5th International Conference on Machine Learning and Computer Application (ICMLCA). Hangzhou: IEEE (2024). p. 619–22. doi: 10.1109/ICMLCA63499.2024.10753879

[36] Huang D Hu Z Wang Z. Performance analysis of Llama 2 among other LLMs. In: 2024 IEEE Conference on Artificial Intelligence (CAI). Singapore: IEEE (2024). p. 1081–5. doi: 10.1109/CAI59869.2024.00108

[37] Saab K Tu T Weng WH Tanno R Stutz D Wulczyn E . Capabilities of gemini models in medicine. arXiv [preprint]. (2024) arXiv:2404.18416.

[38] Curto C Giordano D Indelicato DG Patatu V. Can a llama be a watchdog? Exploring llama 3 and code llama for static application security testing. In: *2024 IEEE International Conference on Cyber Security and Resilience (CSR)*. London: IEEE (2024). p. 395–400. doi: 10.1109/CSR61664.2024.10679444

[39] Aytekin MU Ayhan Erdem O. Generative pre-trained transformer (GPT) models for irony detection and classification. In: 2023 4th International Informatics and Software Engineering Conference (IISEC). Ankara: IEEE (2023). p. 1–8. doi: 10.1109/IISEC59749.2023.10391005

[40] Mohammad F Clark B Hegde R. Large language model (LLM) & GPT, a monolithic study in generative AI. In: 2023 Congress in Computer Science, Computer Engineering, & Applied Computing (CSCE). Las Vegas, NV: IEEE (2023). p. 383–8. doi: 10.1109/CSCE60160.2023.00068

